# Identification of Putative ORF5 Protein of Porcine Circovirus Type 2 and Functional Analysis of GFP-Fused ORF5 Protein

**DOI:** 10.1371/journal.pone.0127859

**Published:** 2015-06-02

**Authors:** Qizhuang Lv, Kangkang Guo, Han Xu, Tao Wang, Yanming Zhang

**Affiliations:** Laboratory of Veterinary Public Health and Food Safety, College of Veterinary Medicine, Northwest A&F University, Yangling, 712100, PR China; Virginia Polytechnic Institute and State University, UNITED STATES

## Abstract

Porcine circovirus type 2 (PCV2) is the essential infectious agent responsible for causing porcine circovirus-associated diseases in pigs. To date, eleven RNAs and five viral proteins of PCV2 have been detected. Here, we identified a novel viral gene within the PCV2 genome, termed ORF5, that exists at both the transcriptional and translational level during productive infection of PCV2 in porcine alveolar macrophages 3D4/2 (PAMs). Northern blot analysis was used to demonstrate that the ORF5 gene measures 180 bp in length and overlaps completely with ORF1 when read in the same direction. Site-directed mutagenesis was used to show that the ORF5 protein is not essential for PCV2 replication. To investigate the biological functions of the novel protein, we constructed a recombinant eukaryotic expression plasmid capable of expressing PCV2 ORF5. The results show that the GFP-tagged PCV2 ORF5 protein localizes to the endoplasmic reticulum (ER), is degraded via the proteasome, inhibits PAM growth and prolongs the S-phase of the cell cycle. Further studies show that the GFP-tagged PCV2 ORF5 protein induces ER stress and activates NF-κB, which was further confirmed by a significant upregulation in IL-6, IL-8 and COX-2 expression. In addition, five cellular proteins (GPNMB, CYP1A1, YWHAB, ZNF511 and SRSF3) were found to interact with ORF5 via yeast two-hybrid assay. These findings provide novel information on the identification and functional analysis of the PCV2 ORF5 protein and are likely to be of benefit in elucidating the molecular mechanisms of PCV2 pathogenicity. However, additional experiments are needed to validate the expression and function of the ORF5 protein during PCV2 infection in vitro before any definitive conclusion can be drawn.

## Introduction

Porcine circovirus type 2 (PCV2), a member of the genus *Circovirus* in the family *Circoviridae*, is now accepted as the primary causative agent of naturally occurring Postweaning Multisystemic Wasting Syndrome (PMWS) [1–4]. PMWS first occurred in Canada in 1991 and has since grown to affect swine populations worldwide [5–6]. This disease primarily affects weanling piglets and is clinically characterized by fever, unthriftiness, respiratory distress, enlarged lymph nodes, and occasional jaundice and diarrhea [7–8]. Many other conditions, including congenital tremors (CT), porcine dermatitis and nephropathy syndrome (PDNS), porcine respiratory disease complex (PRDC), reproduction disorders and fetal myocarditis, are also closely related to PCV2 infection [9–14]. PCV2 infection leads to the development of microscopic lesions mainly dominated by lymphoid depletion and histiocytic infiltration in lymphoid tissues [9, 15–17]. Mortality rates may vary from 15 to 20% and can be significantly higher in the presence of co-infection with either porcine reproductive and respiratory syndrome virus (PRRSV) or *mycoplasma hyopneumoniae* [7–8, 18]. PCV2 infection has thus far caused huge economic losses to the swine industry worldwide.

The PCV2 genome is a single-stranded, ambisense, closed circular DNA containing between 1,766 and 1,768 nucleotides [19–20]. Although the nucleotide homology within individual isolates of either PCV1 or PCV2 is over 90%, the homology between PCV1 and PCV2 as serotypes is only 68% to 76% [19, 21]. This diversity may be responsible for the differences in pathogenicity or virulence observed for these serotypes. The virus was predicted to possess 11 overlapping open reading frames (ORFs) [19], of which four have been characterized in detail in replicating PCV2. On the viral strand, there is the ORF1 (nucleotides 51–995) gene, which encodes the Rep and Rep' proteins essential for PCV2 replication [22]. On the complementary strand, there are the ORF2 (nucleotides 1735–1034), ORF3 (nucleotides 671–357) and ORF4 (nucleotides 565–386) genes, which encode the capsid protein [23], apoptotic protein [21] and controversial anti-apoptotic protein, respectively [24–25]. Previous transcriptional analysis conducted during productive PCV2 infection of porcine kidney cells measured nine unique PCV2-specific RNA transcripts (designated-CR, Rep, Rep′, Rep3a, Rep3b, Rep3c, NS515, NS672, and NS0) [26]; however, only CR, Rep and Rep′ transcripts were proven capable of translation into functional proteins [22, 26–27]. In contrast, ORF3 and ORF4 transcripts, which are not included in the above nine RNAs, were subsequently confirmed to play important roles in the pathogenesis of PCV2; ORF3-deficient PCV2 is attenuated in its natural host [28], and ORF4-deficient PCV2 induces a higher viral load and more severe microscopic lesions in the spleen during early stages of infection in mice [24]. Collectively, these research findings indicate that PCV2 pathogenesis is both complicated and multigenic and that the individual viral proteins encoded by ORF2, ORF3 or ORF4 are insufficient in determining pathogenicity. Previous computational analysis has revealed that the PCV2 genome contains at least seven ORFs with the potential to encode proteins > 5 kDa [29]; to date, four have been identified. However, it has not yet been determined whether the three additional predicted proteins exist or whether there are still other viral proteins encoded by PCV2 that control its pathogenicity.

Sequence alignment analysis of PCV2 isolates from varying geographic regions suggested that the putative ORF5 gene (proposed to exist by Hamel et al) might not encode a functional protein, as translation of the protein was found to terminate after only seven amino acids in some Chinese PCV2 strains [19, 30]. However, several groups have aligned the genomic sequences of various PCV2 isolates and found a region of extremely high identity (over 95%) at the amino acid level, suggesting that PCV2 encodes a novel protein, although different names for this possible protein have been used in the literature (Gen Bank accession no. O92288, Q77GS3, Q77S04, etc). This new putative viral gene (designated ORF5 in this study), which is entirely different from that predicted by Hamel et al in 1998 [19], completely overlaps the ORF1 gene and is read in the same direction. The ORF5 gene is approximately 180 bp (spanning nucleotides 553–732) and encodes a polypeptide that is predicted to be of 6.5 kDa in molecular weight, whereas the corresponding region of PCV1 does not appear to produce a peptide. As PCV1 isolates are naturally nonpathogenic [31–32] and PCV2 isolates are pathogenic, this putative polypeptide may be important for the pathogenesis of PCV2. Presently, no data exist that experimentally confirm the existence of a novel ORF5-encoded protein or its potential biological implications. Porcine alveolar macrophages, one type of porcine monocyte/macrophage lineage cells [9, 33–35], serve as the primary responders of the pulmonary innate immune system and protect against the invasion of various pathogens [36].

In the present study, we identified a novel ORF5 protein that is not essential for PCV2 replication at either the transcription or translation level. We then demonstrated for the first time that the GFP-tagged PCV2 ORF5 protein both prolongs the S-phase of the cell cycle and induces a signaling cascade that results in the activation of NF-κB. This NF-κB activation leads to the upregulation of Interleukin-6 (IL-6), Interleukin-8 (IL-8), and Cyclooxygenase-2 (COX-2). Finally, we uncovered five different cellular proteins that interact with the ORF5 protein.

## Materials and Methods

### Virus, cells, sera and animals

The wild-type PCV2 Yangling strain (wPCV2) [37] isolated from a pig with naturally occurring PMWS was propagated in PK-15 cells (ATCC: CCL-33) (Kindly provided by Dr. Qing-hai Tang, the Harbin Veterinary Research Institute) [38]. The porcine alveolar macrophages 3D4/2 (PAMs) (ATCC: CRL-2845) were grown in RPMI 1640 medium (Gibco, UK) as described by Weingartl et al in 2002 [39]. PAMs were cultured in medium containing PCV2 at an MOI of 1.0. PCV-free PK-15 cells were cultured in high glucose Dulbecco’s-modified Eagle’s medium (DMEM) (Gibco, UK) supplemented with 10% FBS. Specific pathogen-free (SPF) BALB/c mice weighing approximately 20 grams each were purchased from the The Fourth Military Medical University, Chinese People's Liberation Army, Xi'an, China. All animal procedures were approved and supervised by the Animal Care Commission of the College of Veterinary Medicine, Northwest Agriculture & Forestry University. Every effort was made to minimize animal pain, suffering and distress and to reduce the number of animals used.

### RT-PCR and Quantitative Real-time PCR

Total cell RNA was isolated using Trizol (Invitrogen, USA) and reverse transcription of the RNA was conducted using the PrimeScript RT reagent Kit with gDNA Eraser (TAKARA, China), which removed any contaminating viral DNA. To detect whether the ORF5 gene could express at a transcriptional level in PCV2-infected cells, PCR was carried out by using the F553 (5ˊ-ATGTACACGTCATTGTGGGG-3ˊ) / R732 (5ˊ-TCAGTAGATCATCCCAGGGCAGC-3ˊ) primer pair. The resultant PCR product was electrophoresed in 1% agarose gel and photographed. To determine the targeted cDNA or viral DNA, quantitative real-time PCR was conducted using SYBR *Premix Ex Taq* II (Tli RNaseH Plus) (TaKaRa) following the manufacturer’s recommendations. Reactions were performed on an iQ5 Multicolor Real-Time PCR Detection System (Bio-Rad, USA). RNA expression was normalized by quantification of porcine β-actin as a housekeeping gene. Relative transcript levels were analyzed by using the formula of 2-delta-delta Ct described by Livak and Schmittgen [40].

For the standard curve, serial 10-fold dilutions of recombination plasmids pSK-W and p19T-β (β-actin gene cloned into PMD19-T) were used to test sensitivity and efficiency of the assay. Each assay was carried out in triplicate and only standard curve with coefficient of correlation (*R* ^2^) upon 0.99 was accepted.

### Northern blot

To identify the ORF5 transcript, northern blot was performed as previously described with slight modifications [24]. Briefly, the digoxigenin (DIG)-labeled ORF5 double strand DNA probe (probe D) was generated using the PCR DIG Probe Synthesis Kit (Roche, Switzerland) with primers (F 5ˊ-ATGTACACGTCATTGTGGGGC-3ˊand R 5ˊ-TCAGTAGATCATCCCAGGGCAGC-3ˊ) and pMD-19T-ORF1 as the PCR template. PAMs infected with wPCV2 were subjected to total RNA extraction using TRIzol reagent and treated with gDNA Eraser. The total RNA was then separated by electrophoresis and transferred to a Hybond-N+ nylon membrane (Amersham, Sweden). After hybridization with a DIG-labeled ORF5 DNA probe, an anti-DIG antibody conjugated to alkaline phosphatase (Roche) was applied to the membrane. Finally, the hybridized bands were visualized using CSPD (Roche) with X-ray and compared to positive bands of known immobilized molecular weights.

### Generation of antibody against ORF5 protein

To facilitate raising antibodies against ORF5, *Eco*RI and *Xho*I sites were engineered into the PCR oligonucleotides used for amplification of the full-length ORF5 gene according to the archived PCV2 Yangling strain nucleotide sequence (S1 Fig) using the following primers: forward, 5ˊ-GCGAATTCATGTACACGTCATTGTGGGG-3ˊ, and reverse, 5ˊ-GGCCTCGAGTCAGTAGATCATCCCAGGGCAGC-3ˊ. PCR products were then inserted into the prokaryotic expression vector pGEX-6p-1 (Amersham). The recombinant plasmid (pGEX-ORF5) was sequenced and transformed into *Escherichia coli* BL21 cells (Invitrogen). These cells were induced to express ORF5 using isopropylthio-β-D-galactoside (Amersham). The purified GST-ORF5 protein was injected into SPF BALB/c mice to induce polyclonal antibody production as previously described [24]. The reactivity of the anti-ORF5 pAb was confirmed via western blot and indirect immunofluorescence assay (IFA), performed on either ORF5-transfected- or wPCV2-infected PK-15 cells.

### Western blot and ELISA

Cells were harvested and treated with lysis buffer for 30 min on ice. Protein samples were separated by 12% SDS-PAGE and transferred onto PVDF membranes (Millipore, USA). The membranes were blocked with 5% skim milk and then incubated with indicated primary antibodies at 37°C for 2 h, followed by HRP-conjugated secondary antibodies. The cellular protein β-actin was additionally detected to serve as an internal control. Immunoreactive bands were visualized using an enhanced chemiluminescence (ECL) western blotting analysis system (Thermo, USA). Secreted IL-6, IL-8 and COX-2 proteins, obtained from cell culture supernatants of PAMs transfected with pEGFP-ORF5 or pEGFP-C1 at 48 h post infection, were measured using swine IL-6, IL-8 and COX-2 ELISA kits (Cusabio, China) according to the manufacturer’s protocol. Additionally, changes to NF-κB activity in PAMs transfected with either the GFP-ORF5 protein or GFP alone were monitored using the TransAM NF-κB transcription factor assay kit (Active Motif, USA) following the manufacturer’s instructions.

### IFA

Indirect immunofluorescence assay was conducted according to previous protocols with some modifications [41]. Briefly, cells in 96-well plates were infected with either wild-type PCV2 or recovered viruses at an MOI of 1.0. Following incubation at 37°C with 5% CO_2_ for 72 h, cells were fixed with 4% paraformaldehyde in PBS at room temperature for 20 min and subsequently permeabilized with PBS buffer containing 0.25% Triton X-100 and 5% dimethyl sulfoxide for 10 min. Thereafter, the cells were blocked using PBS with 5% skim milk at 37°C for 1 h and stained for 1 h at 37°C with the primary mouse anti-ORF5 polyclonal antibodies (pAb) or the rabbit anti-Cap pAb diluted in PBS-T with 1% bovine serum albumin. After washing with PBS, the cells were incubated with fluorescein isothiocyanate-conjugated goat anti-mouse or anti-rabbit IgG (Sigma-Aldrich, USA). Finally, the cells were incubated with Hoechst33342 (Beyotime) at room temperature for 10 min. After rinsing with distilled water and supplementation with fluorescence mounting medium, images were obtained using laser confocal scanning microscopy (Model LSM510 META, Germany). Mock-infected PAMs were used as a control.

### Construction of PCV2 mutant and recombinant plasmids

**Fig 1:**
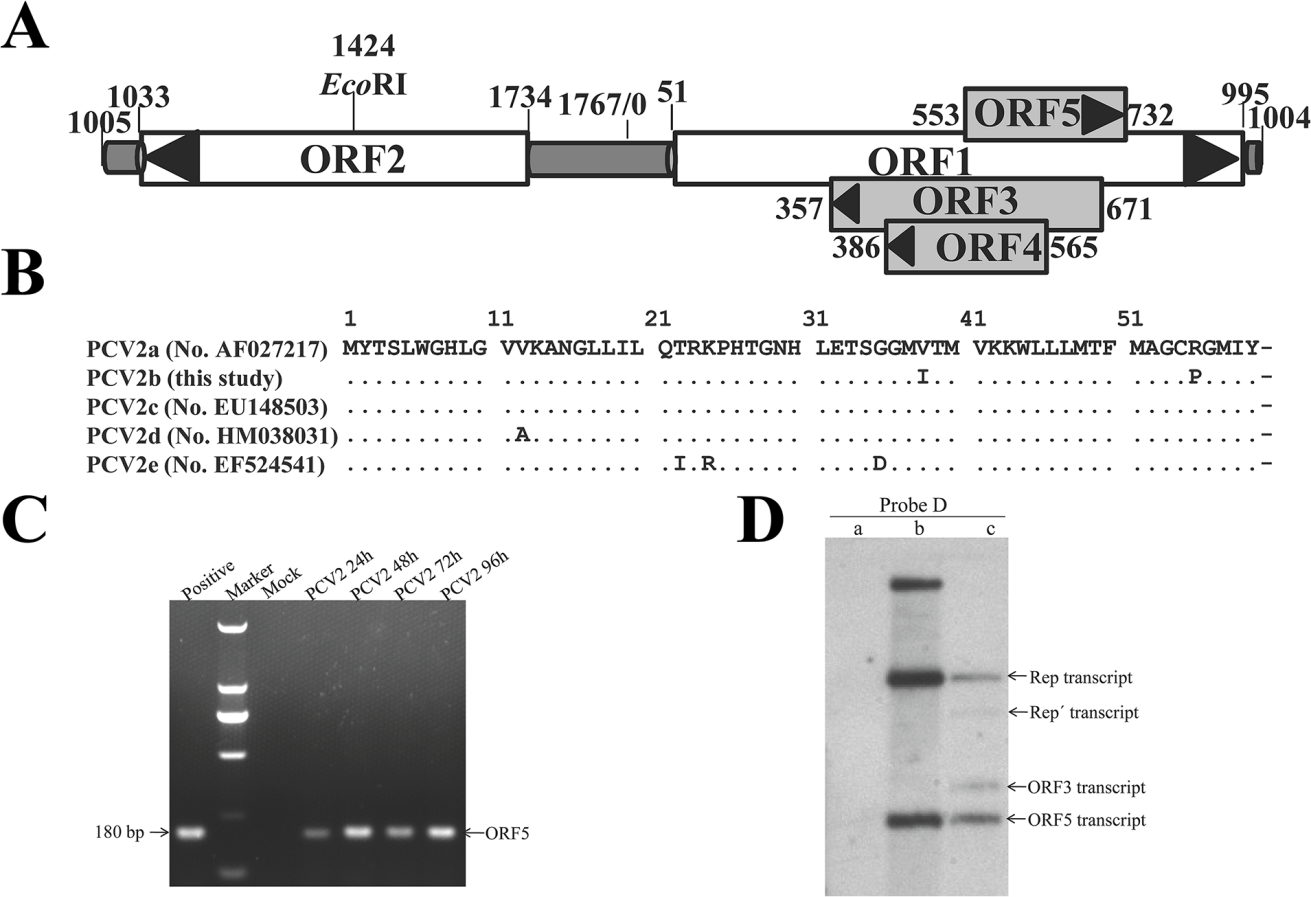
Genomic location of ORF5 gene and Detection of ORF5 transcripts by RT-PCR and Northern blot from PCV2-infected cells. (A) Genomic schematic of PCV2 Yangling strain (wPCV2). Coding sequences of the five ORFs are annotated with nucleotide coordinates that indicate the nucleotide site of each gene. The ORF2, ORF3 and ORF4 genes are transcribed leftward, while the ORF1 and ORF5 genes are transcribed rightward. The *Eco*RI restriction enzyme site is also indicated. (B) Nucleotide and amino acid alignments of the putative ORF5 reported by Hamel et al in 1998 (pmws, GenBank accession no. AF027217 [19]) and our team (wPCV2, this study) using the ClustalV Method. Homologous nucleotides and amino acids are indicated by asterisks. (C) Analysis of the ORF5 gene in PCV2-infected cells by RT-PCR. RNA was isolated from PCV2- or mock-infected cells and copied into cDNA. The cDNA was amplified with a pair of ORF5 primers. Positive fragment was amplified from the PCV2 genome using PCR. (D) Northern blot identification of the ORF5 transcript in PAMs infected with wPCV2. Total RNA samples were isolated from mock- and wPCV2-infected PAMs. Lane a, no RNA signal was detected in mock-infected PAMs after hybridization with a DIG-labeled ORF5 DNA probe (probe D). Lane b, mixtures of pMD-19T-ORF1 (3625 bp), PCR products of ORF1 (945 bp) and ORF5 (180 bp) were hybridized with probe D. Lane c, Rep, Repˊ, ORF3 and ORF5 transcripts were hybridized with probe D in wPCV2-infected PAMs.

Using the PCV2 Yangling strain as a genomic template (Fig 1), the infectious ORF5-deficient PCV2 mutant clone (pSK-PCV2Δ) was constructed via a Site-Directed Gene Mutagenesis Kit (Beyotime, China). The introduced mutation changed the ORF5 initiation codon from ATG to ACG and did not affect any other aspect of the amino acid sequence of the ORF1 protein. Briefly, amplification of the complete genome of PCV2 with an overlapping region containing the unique *Eco*RI restriction enzyme site (S1 Fig) was accomplished by using Phusion High-Fidelity PCR master mix (Roche) with a pair of primers (F-*Eco*RI, 5ˊ-CGGAATTCAACCTTAACCTTTCTTATTC-3ˊ and R-*Eco*RI, 5ˊ-CGGAATTCTGGCCCTGCTCCCCGATCACCCAGG-3ˊ). After separation by gel electrophoresis and purification by using a PCR cleanup kit (Axygen, USA), the genomic PCR fragment of expected size was digested with *Eco*RI and subcloned into the pBluescript SK+ (pSK) vector (Stratagene, USA) to generate the recombinant plasmid pSK-W. After confirmation by restriction analysis and DNA sequencing, the mutant plasmid pSK-PCV2Δ was constructed with a set of mutagenesis primers (5ˊ-GATTGGAAGACTAACGTACACGTCATTG-3ˊ and 5ˊ-CAATGACGTGTACGTTAGTCTTCCAATC-3ˊ) (Underlined letters show the mutant site) following the manufacturer’s guide. To obtain the corresponding infectious clones, the full-length wild-type and mutant PCV2 genomes were excised from the pSK plasmid using the restriction enzyme *Eco*RI and re-circularized by ligation with T4 DNA ligase overnight at 16°C. TurboFect Transfection Reagent (Thermo) was used to transfect the resultant DNA mixture into PCV-free PK-15 cells that were cultured overnight, and 300 mM D-glucosamine was added to the culture media at 24 h post-transfection as previously described [42]. At 72 h after transfection, the viral stocks were harvested from the total lysates of genome-transfected cells.

To prepare the eukaryotic expression vector of ORF5, the coding sequences of ORF5 gene were PCR-amplified from PCV2 genomic DNA using the following primers: forward, 5ˊ-CGGAATTCTATGTACACGTCATTGTGGGG-3ˊ, and reverse, 5ˊ- ATTGGATCCTCAGTAGATCATCCCAGGGCA-3ˊ. After purification, the *Eco*RI/*Bam*HI fragment of ORF5 was directionally cloned into the mammalian expression vector pEGFP-C1 (Clontech). The recombinant plasmid was sequenced and named pEGFP-ORF5.

### Transfection and infection

Transient expression experiments were conducted to verify the expression of PCV2 ORF5 *in vitro*. PAMs were cultured in 6-well plates and transfected with either GFP vector only or GFP-ORF5 using TurboFect Transfection Reagent (Thermo) according to the manufacturer’s instructions. At 48 h after transfection, ORF5 protein expression was determined by western blot analysis using the mouse anti-ORF5 polyclonal antibody.

To test viral infectivity, the collected total lysates from genome-transfected PK-15 cells were used to infect PCV-free PAMs at an MOI of 1.0. Following treatment with glucosamine as described above, the infectivity of recovered virus was determined via IFA using antiserum against either the ORF5 antibody or the anti-Cap pAb after infection.

### Assays for virus replication and viral mRNA expression levels

To study the replicative ability of rescued PCV2, confluent PAMs were inoculated with wPCV2, rPCV2, or PCV2Δ at an MOI of 1.0, and the cell monolayers were harvested at different time intervals post-inoculation followed by three freeze-thaw cycles. Viral DNA copies were quantified via quantitative real-time PCR assay using primers F1150 (5ˊ-GTGGTCCACATTTCCAGCAGTT-3ˊ) and R1481 (5ˊ-GCTCAAACCCCCGCTC-3ˊ). Viral titer was also determined via the end-point dilution assay using tissue culture infective doses as described by Reed and Muench [43].

Total RNA was extracted from virus-infected PAMs using Trizol reagent as previously described. The recovered RNA samples were incubated with gDNA Eraser at 42°C for 2 min to remove any contaminating viral DNA and were then treated with a reverse transcriptase enzyme mix at 42°C for 15 min to produce cDNA. The expression of the ORF1, ORF2, ORF3, ORF4 and ORF5 mRNAs were analyzed by quantitative RT-PCR using five primer pairs (summarized in Table 1). Simultaneously, the primers Fβ-actin and Rβ-actin were used to amplify a segment of porcine β-actin to serve as an internal control.

**Table 1:** Sequences of primer pairs used for qRT-PCR.

Gene	Forward Primer(5`-3`)	Reverse Primer(5`-3`)	Product(bp)	Accession no.
ORF1	CATCGAGAAAGCGAAAGG	CTCCATCAGTAAGTTGCCTTC	73	AY188355
ORF2	GTAAACTACTCCTCCCGCC-	CCACATTTCCAGCAGTTTG	157	AY188355
ORF3	GTCTTCCAATCACGCTTCTG	GGACAACGGAGTGACCTG	161	AY188355
ORF4	ACAAGGTACTCACAGCAG	TGGAGTGTGGAGCTCCTAG	66	AY188355
ORF5	ATGTACACGTCATTGTGGGG	TCAGTAGATCATCCCAGGGC	180	AY188355
IL-6	GAGCCCACCAGGAACGAAAGAG	GCAGTAGCCATCACCAGAAGCA	295	AM501528
IL-8	GGCTGTTGCCTTCTTG	TGGAATGCGTATTTATG	109	M86923
COX-2	TTCAACCAGCAATTCCAATACCA	GAAGGCGTCAGGCAGAAG	87	AF207824
Cyclin A	AAGTTTGATAGATGCTGACCCGTAC	GCTGTGGTGCTCTGAGGTAGGT	194	GQ265874
GRP78	AATGGCCGTGTGGAGATCA	GAGCTGGTTCTTGGCTGCAT-	114	X92446
Bax	ATGATCGCAGCCGTGGA	GGGCCTTGAGCACCAGTTT	139	AJ606301
Bcl-2	TTGTGGCCTTCTTTGAGTTCG	CTACCCAGCCTCCGTTATCC	150	XM_003121700.1
β-actin	CAAGGACCTCTACGCCAACAC	TGGAGGCGCGATGATCTT	130	DQ845171.1

### Cell proliferation assay

To determine the growth properties of cells expressing the PCV2 ORF5 protein and control cells, the cell proliferation assay was performed using Cell Counting Kit-8 (CCK8/WST-8) (Dojindo Laboratories, Japan) following the manufacturer’s protocol.

### Flow cytometry

To measure the impact that the PCV2 ORF5 protein has on the cell cycle, cells transfected with either pEGFP-ORF5 or pEGFP-C1 for 48 h were fixed with 75% ethanol for 3 days at 4°C and were then incubated with propidium iodide (PI) in the dark for 0.5 h. A set of control cells was also subjected to the same experimental conditions. Finally, the nuclear DNA content was tested by a Coulter Epics XL flow cytometer (Beckman, USA) as previously described [44].

Flow cytometry was also conducted to determine the effects that the PCV2 ORF5 protein has on apoptosis, using the BD Pharmingen PE Annexin V Apoptosis Detection Kit I (Becton Dickinson, USA) according to the manufacturer’s recommendations. Briefly, PAMs grown in T25 flasks were transfected either with pEGFP-ORF5 or pEGFP-C1. After incubation at 37°C with 5% CO_2_ for 48 h, the cells were treated with trypsin without EDTA and were resuspended to final concentration of 1 × 10^5^ cells/ml. After the addition of 5 μl of PE Annexin V and 5 μl of 7-AAD, the cells were incubated for 15 min at room temperature in the dark and analyzed using the flow cytometer. Data analysis was performed using the CXP Software (Beckman) on the basis of the FSC-SSC (forward light scatter-side scatter) dot plot.

### Confocal microscopy

To examine the expression and subcellular localization of the PCV2 ORF5 protein, PAMs grown on glass bottom dishes (35 mm) were transfected with either pEGFP-ORF5 or pEGFP-C1. Following incubation for 48 h at 37°C, both the cells expressing the GFP-ORF5 protein and the control cell groups (either PAMs expressing GFP alone or cells that were not transfected) were incubated with Hoechst33342 (Beyotime) at room temperature for 10 min and then incubated with ER-Tracker Red probe (Invitrogen) for 15 min at 37°C. Images were obtained using laser confocal scanning microscopy (Model LSM510 META, Germany) and were processed by Adobe Photoshop CS6 software (Adobe, USA).

### Observation of protein degradation characteristics

PAMs grown in 6-well plates were transfected with either pEGFP-ORF5 or pEGFP-C1. After incubation for 24 h at 37°C, the cells were treated with 10 μM MG132, 30μM BAPTA-AM, 20 mM NH_4_Cl, or 50 μM E64, respectively. Another 12 h later, the cells were harvested for Western blot analysis.

### Yeast two-hybrid screen

To perform the yeast two-hybrid screen, the Matchmaker GAL4 Two-Hybrid system 3 (Clontech) was used as previously described [45]. A nuclear localization signal (PKKKRKV) was added to the N-terminal of the ORF5 protein to meet the requirements of the yeast two-hybrid model. The Y2H yeast strain was transformed simultaneously with pGBKT7-ORF5 and the prey plasmid pGADT7, which contained a PAM cDNA library of 2 × 10^6^ clones. Plasmids were rescued from colonies grown on quadruple dropout plus X-α-Gal (QDO/X) plates, which lacked adenine, histidine, tryptophan and leucine, and were sequenced after their activity was confirmed by co-transformation of the respective bait and prey plasmids. Identity of the positive clones was obtained from database searches using the NCBI BLASTP program.

### Other reagents

The rabbit anti-Cap polyclonal antibody (pAb) was a kind gift from Prof. Zeng-Jun Lu, Lanzhou Veterinary Research Institute, Chinese Academy of Agricultural Sciences. Monoclonal antibodies against cyclin A (CycA), GRP78, IκBα, NF-κB p65 and β-actin were purchased from Santa Cruz Biotechnology (USA). 1,2-bis(*o*-aminophenoxy)ethane-*N*,*N*,*N′*,*N′*-tetraacetic acid tetra(acetoxymethyl)ester (BAPTA-AM), MG132, *trans*-epoxysuccinyl-L-leucylamido-4-guanidino butane (E64) and ammonium chloride (NH_4_Cl) were purchased from Sigma-Aldrich (USA). The plasmid pMD-19T-ORF1 (our unpublished construct) (Takara) was maintained in our laboratory.

### Statistical analysis

Results are presented as mean ± the standard deviations (SD). Statistical comparisons were made by using Student’s *t*-test. *p* value less than 0.05 was considered statistically significant and *p* value less than 0.01 was considered highly significant.

## Results

### Identification of ORF5 transcription within PCV2-infected cells

To confirm the expression of ORF5 at the transcriptional level, total cellular RNA extracted from wPCV2-infected and mock-infected PAMs were subjected to RT-PCR and northern blot hybridization. Fig 1 demonstrates that the amplification products of RT-PCR at the indicated time points were genetic fragments of the expected size, suggesting that the ORF5 gene could be transcribed from the PCV2 genome. To confirm these results, the extracted total RNA was hybridized with a DIG-labeled ORF5 DNA probe. After hybridization with DNA probe D, a total of four RNA bands of approximately 950 bp, 750 bp, 300 bp and 180 bp were found (Fig 1), which were successively identical to the Rep, Repˊ, ORF3 and putative ORF5 transcripts in length, respectively. No hybridized band was detected in mock-infected PAMs (Fig 1). The results further indicated that the ORF5 mRNA could be abundantly detected in PCV2-infected cells.

### Expression of the ORF5-encoded protein within PCV2-infected cells

**Fig 2:**
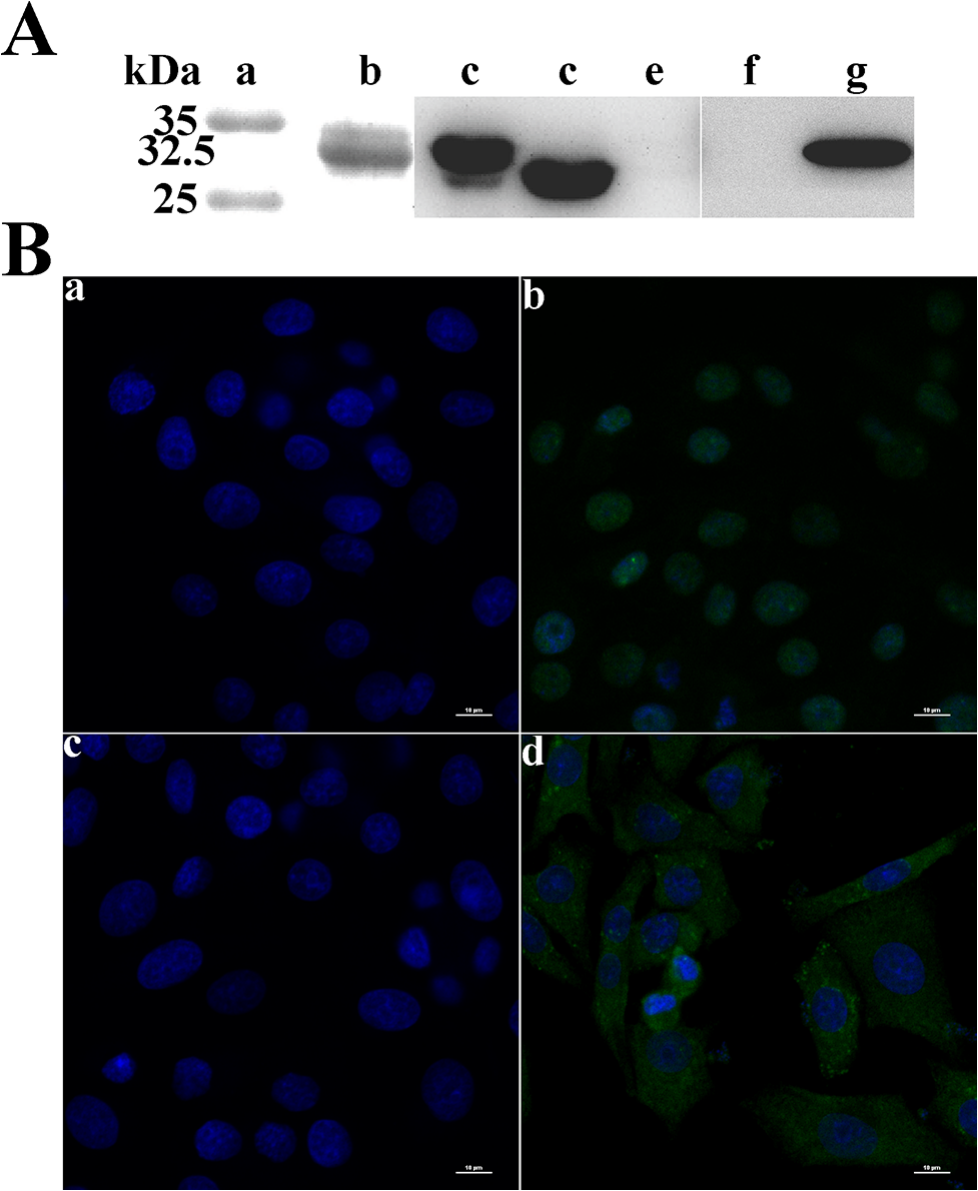
Confirmation of the expression of the PCV2 ORF5 protein within PCV2-infected cells. (A)Western blot analysis of ORF5 protein expressed in bacterial cells or PAMs. Lysed samples were electrophoresed on15% SDS-PAGE gels, transferred onto nitrocellulose membranes and probed with specific antibodies. Lane a, protein molecular weight marker. Lane b, the GST-ORF5 protein purified from E. coli was detected by the pAb against PCV2 ORF5 protein. Lane c to e, the lysates of GST-ORF5-expressing, GST-expressing and blank E.coli were detected by rabbit anti-GST pAb respectively. Lane f, pEGFP-C1-transfected PAMs were probed with the pAb against PCV2 ORF5 protein. Lane g, pEGFP-ORF5-transfected PAMs were probed with the pAb against PCV2 ORF5 protein. (B) IFA analysis of PAMs infected with wide-type PCV2. At 48 h after infection, the cells were fixed and labeled with the rabbit anti-Cap pAbs (panel b) or mouse anti-ORF5 pAbs (panel d) for confocal microscopic analysis. As negative controls, mock-infected cells were also probed with the rabbit anti-Cap pAbs (panel a) or mouse anti-ORF5 pAbs (panel c). Nuclei were stained with Hoechst 33342 (bars, 10 μm).

To identify the expression of the ORF5 protein at the translational level, mouse polyclone antibodies (pAbs) against ORF5 were produced. As shown in Fig 2, the generated pAbs could specifically recognize the viral ORF5 protein expressed from either *E*. *coli* transformed with the pGEX-ORF5 vector or from PAMs transfected with the pEGFP-ORF5 plasmid. No immunoreactive signal was observed between the pAbs and ORF1-encoded protein expressed from either *E*. *coli* or PAMs (data not shown). To verify whether the ORF5 protein was expressed in replicating PCV2, PAMs were infected with wPCV2 or PCV2Δ as described in the Materials and Methods. IFA analysis was used to demonstrate that the PCV2 ORF5 protein initiates expression during the early stage of infection (data not shown) and peaks at 48 h post-infection (hpi) (Fig 2). Positive control cells expressing the PCV2 Cap protein were also analyzed at 48 hpi and no signals were detected in either mock-infected cells (Fig 2) or in PCV2Δ-infected PAMs (data not shown). Thereafter, total proteins were further harvested from PAMs at the indicated time points post-infection and analyzed by western blotting. Unfortunately, the ORF5-specific mouse pAbs could not detect a signal for the ORF5 protein in wPCV2-infected PAM lysates that were treated with reducing loading buffer and boiled (data not shown). This phenomenon is similar to that observed for the PCV2 ORF4 protein, which also eludes confirmation via immunoblot analysis [24], although the specific reasons behind this remain unknown.

### The ORF5 protein is not essential for viral replication

**Fig 3:**
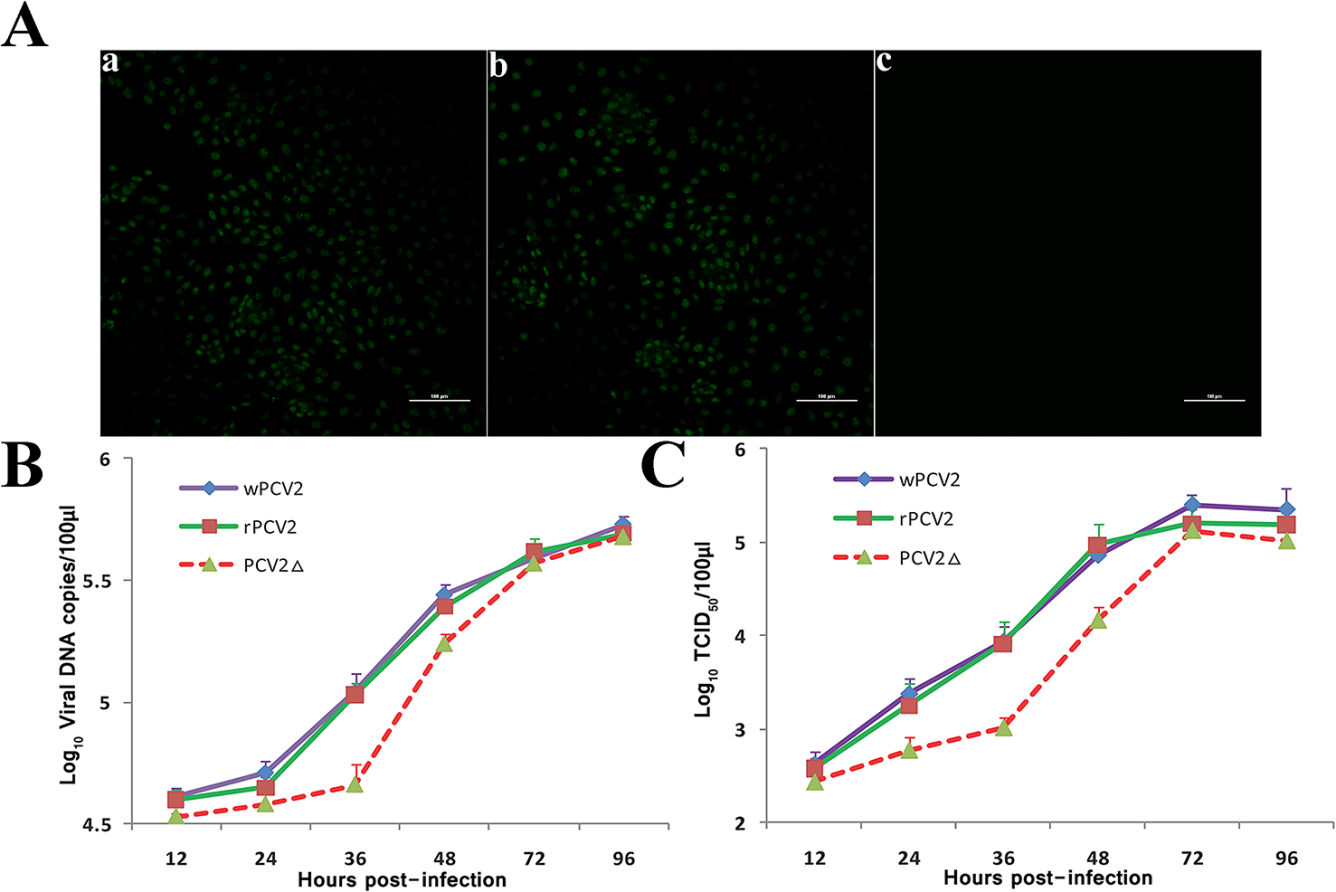
The impact that the lack of ORF5 has on the PCV2 replication. (A) PK-15 cells transfected with either recombinant PCV2 or mutant PCV2 DNA were detected by IFA. (a) Cells transfected with rPCV2 DNA were detected using anti-Cap pAbs, (b) Cells transfected with PCV2Δ DNA were detected using anti-Cap pAbs, (c) Cells transfected with PCV2Δ DNA were detected using anti-ORF5 pAbs. Bar = 100 μm for all the Figures. (B) PCV2-infected PAMs were assayed for viral DNA copy number by real-time PCR. (C) The cell cultures were harvested and PCV2 titers were detected as TCID_50_. Results are presented as mean ± SD (n = 3).

To assess the effect that the PCV2 ORF5 protein has on viral replication, the initiation codon of the ORF5 gene was mutated to generate an ORF5-deficient PCV2 infectious DNA clone, which was then used to infect PK-15 cells. As shown in Fig 3, PK-15 cells transfected with either recombinant PCV2 or mutant PCV2 DNA both generated a viable virus (rPCV2 or PCV2Δ) and only the Cap protein could be detected when PCV2Δ-infected PK-15 cells were analyzed by IFA using the anti-Cap pAb or the anti-ORF5 pAb. No fluorescence was detected in the mock-infected cells using anti-Cap rabbit serum (data not shown). After three rounds of propagation in PK-15 cells, the recovered viruses were then used to measure the replication kinetics of wPCV2, rPCV2 and PCV2Δ via qRT-PCR and IFA. PAMs infected with each virus were harvested at 12, 24, 36, 48, 72 and 96 h post-infection, and viral DNA and virus titers were quantified by real-time PCR and IFA, respectively. As shown in Fig 3, the curves of viral DNA copies at the indicated time points were roughly identical to that of infectious titers throughout the course of infection, and the replicative abilities of wPCV2 and rPCV2 were similar to one another. However, PCV2Δ replicated at a somewhat reduced rate prior to 36 hpi (P < 0.05) and displayed growth characteristics similar to those measured for wPCV2 and rPCV2 at later time points. At 72 hpi, the titers of PCV2Δ, rPCV2 and wPCV2 reached 10^5.12^ TCID_50_/ml, 10^5.2^ TCID_50_/ml and 10^5.4^ TCID_50_/ml, respectively. These results suggested that the lack of ORF5 protein expression might block viral replication during the early stages of infection, although it is not essential for PCV2 replication in cell culture.

### Expression of ORF1, ORF2, ORF3, ORF4, and ORF5 at the transcriptional level

**Fig 4:**
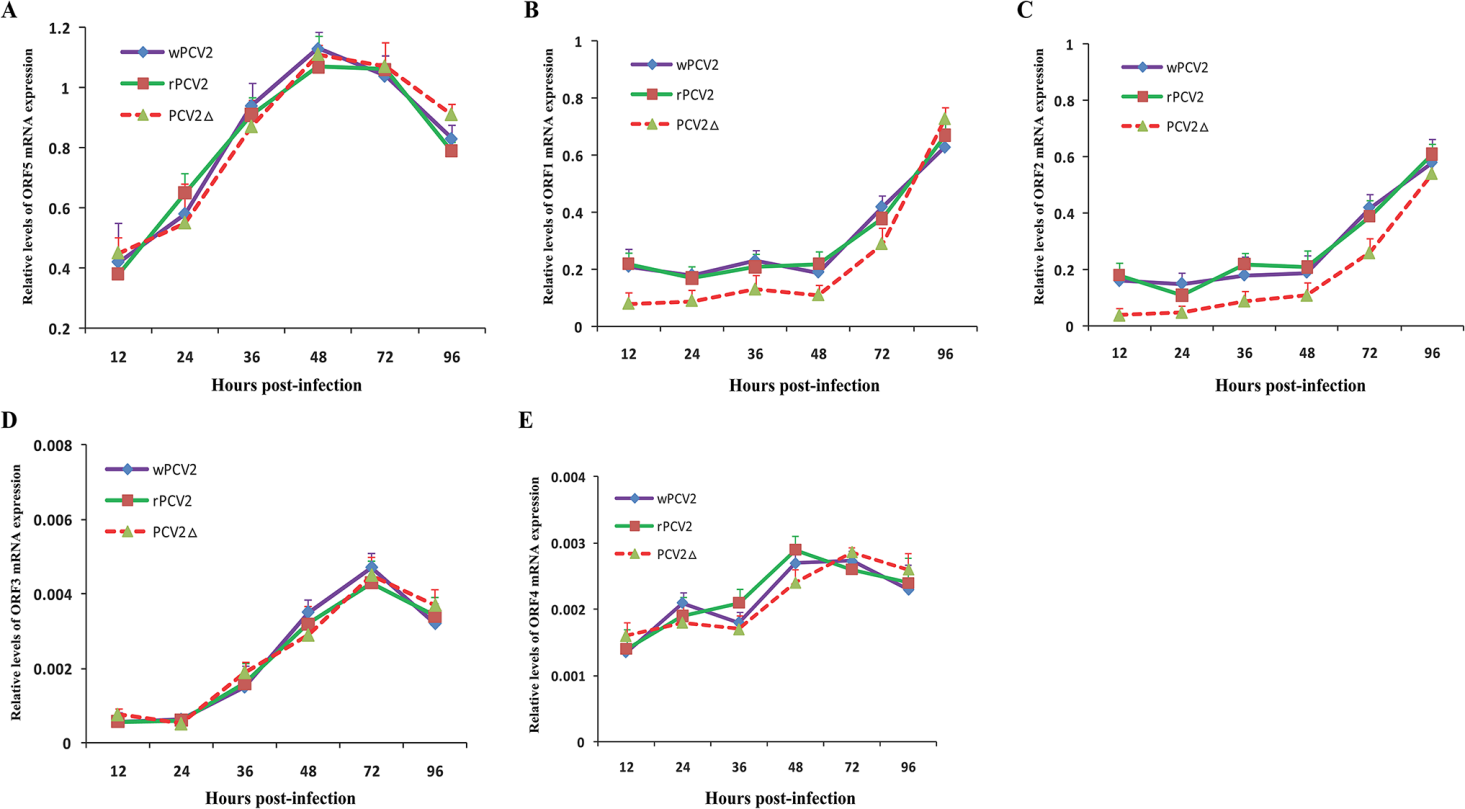
mRNA expression curves. PAMs were infected with wild-type PCV2 (wPCV2), recombinant PCV2 (rPCV2), or a start codon mutant PCV2 (PCV2Δ) at an MOI of 1.0. Cellular total mRNA was harvested at six time points from 12 to 96 h post infection, treated with gDNA eraser and tested by real-time quantitative RT-PCR. (A) Expression of ORF5 mRNAs. (B) Expression of ORF1 mRNAs. (C) Expression of ORF2 mRNAs. (D) Expression of ORF3 mRNAs. (E) Expression of ORF4 mRNAs. Results are presented as group mean cDNA numbers ± SD (n = 3).

The mRNA copies of ORF1, ORF2, ORF3, ORF4 and ORF5 were examined by qRT-PCR. PAMs infected with wPCV2, rPCV2 or mutant PCV2Δ at an MOI of 1.0 were harvested at different times post-infection, and total cellular mRNA was isolated and quantified. Fig 4 depicts the transcription curve of each gene at the indicated time points following infection. The expression curve of ORF5 mRNA demonstrates that the site-directed mutation within ORF5 did not affect its transcription, as the PCV2Δ ORF5 transcript displayed a similar expression curve to wPCV2 or rPCV2 during this experiment (Fig 4). These results suggest that the ORF5 gene may initiate transcription at a more upstream site. However, the expression levels of ORF1 and ORF2 transcripts in PCV2Δ-infected cells were significantly lower than those measured in wPCV2- or rPCV2-infected cells from 12 hpi to 72 hpi (P < 0.05) (Fig 4), suggesting that the lack of ORF5 reduced the transcriptional capacity of ORF1 and ORF2 during the first 72 hours after infection. These results indicate that the replication capacity of ORF5-deficient PCV2 might be smaller than previously thought. Additionally, the expression levels of ORF1 and ORF2 mRNAs were basically in accordance with each other and exactly conformed to the growth rhythm of the virus.

Given the important role of ORF3- and ORF4-associated proteins during PCV2-induced apoptosis, the expression levels of ORF3 and ORF4 mRNAs at different hpi were also examined. It was determined that both the ORF3 and ORF4 transcripts showed an expression curve similar to wPCV2 and rPCV2 (Fig 4), indicating that the ORF5 protein has no impact on the transcription of ORF3 and ORF4 mRNA. Whether this finding can be used to imply that there are no interactions between ORF5 and ORF3 or ORF4 is unclear.

### The GFP-tagged PCV2 ORF5 protein inhibits cell proliferation via prolongation of the S-phase of the cell cycle

**Fig 5:**
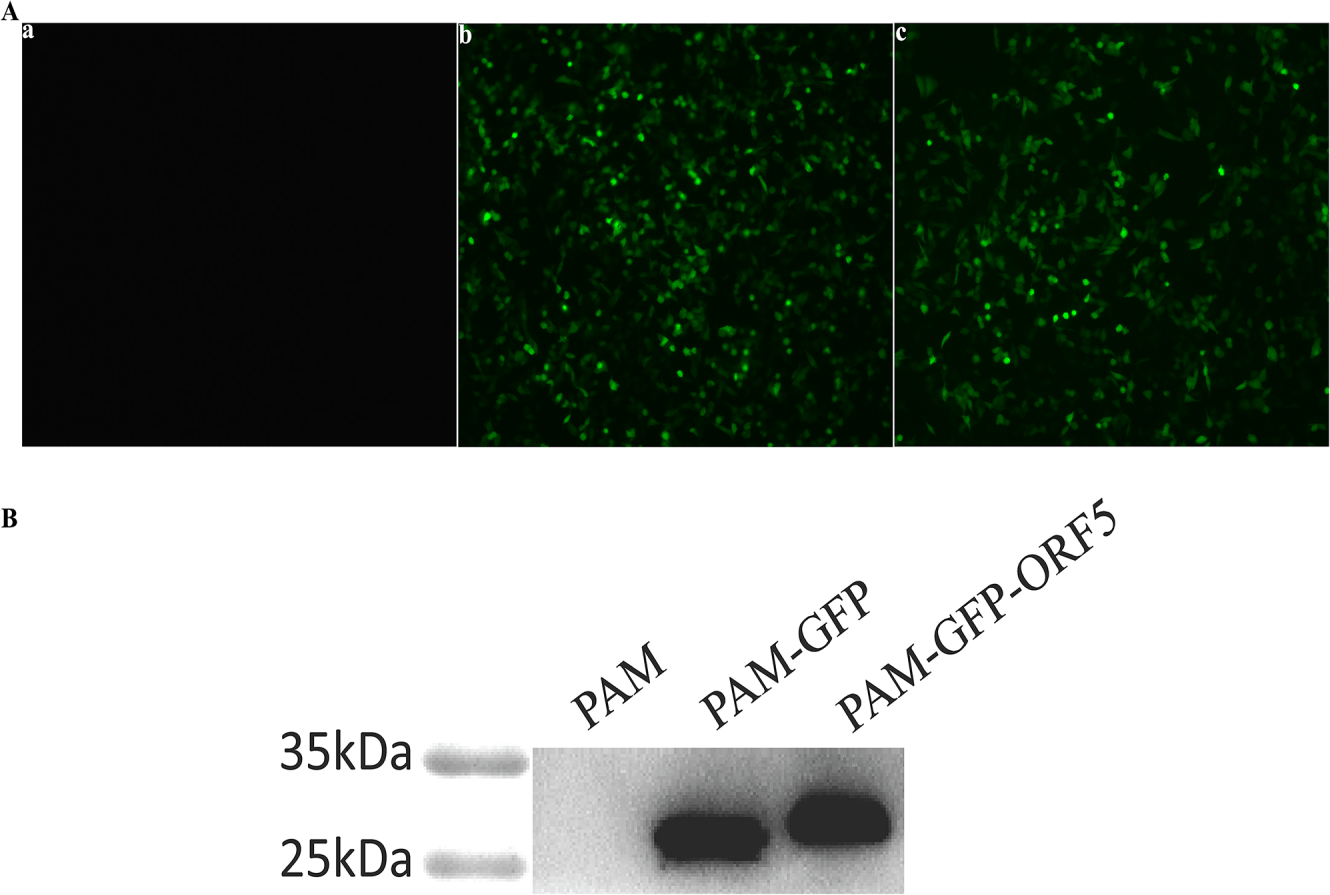
Fluorescence detection and Western blot analyses of expression products of GFP-ORF5 fusion protein in PAMs. (A) The cells were observed under an inverted fluorescence microscope (100×). Panel a, mock-transfected PAMs. Panel b, pEGFP-C1- transfected PAMs. Panel c, pEGFP-ORF5- transfected PAMs. (B) Proteins were isolated from whole extracts of the cells expressing GFP-ORF5 protein and control cells, and then subjected to Western blot using anti-GFP antibody.

**Fig 6:**
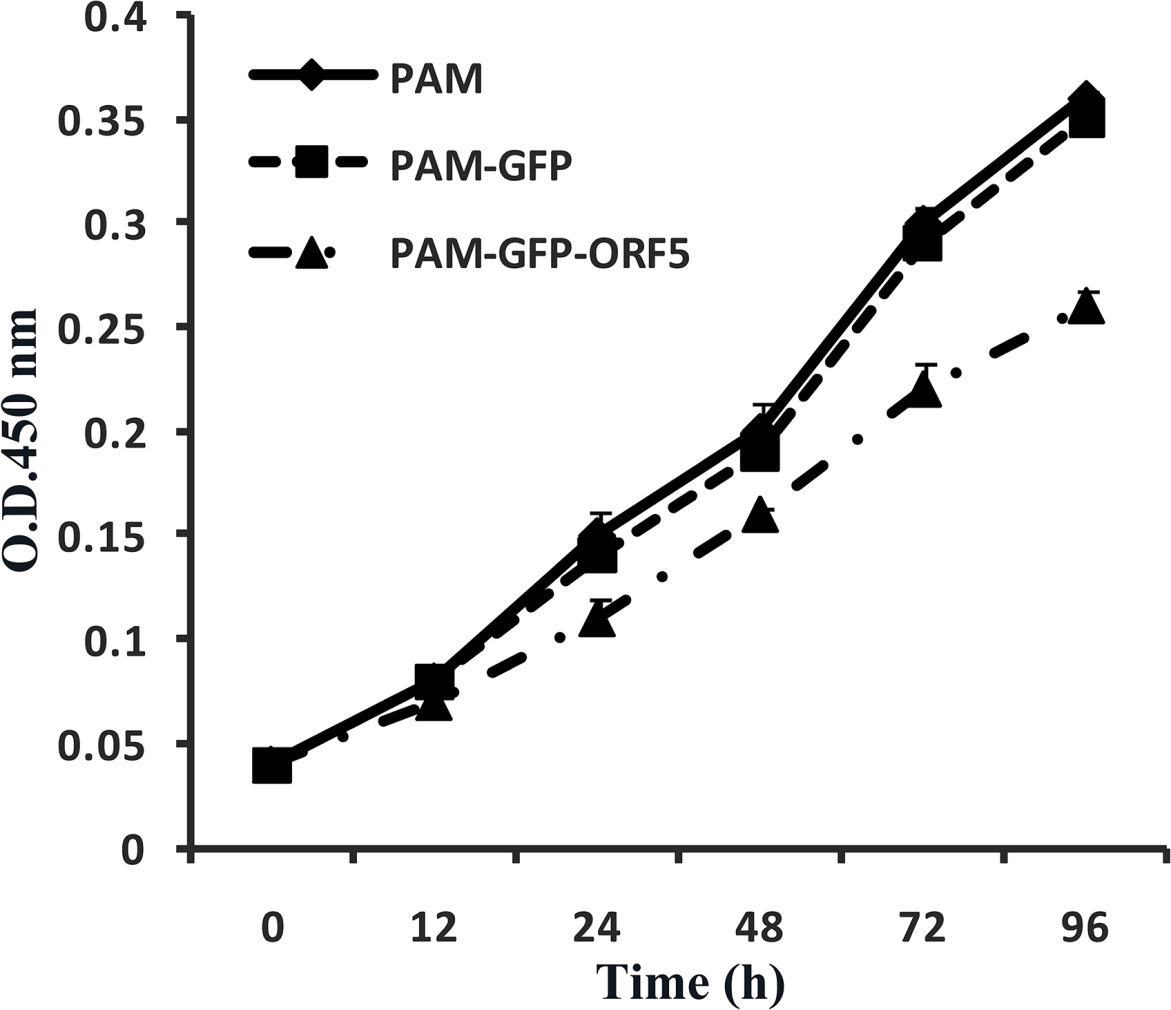
Cell proliferation assays of the GFP-ORF5 protein expressing cells. The CCK8 assay was used to measure proliferation of 3 × 10^3^ cells from PAMs transfected with pEGFP-ORF5 and control cells over time. Each data set represents the mean ± SD of three replicates.

**Fig 7:**
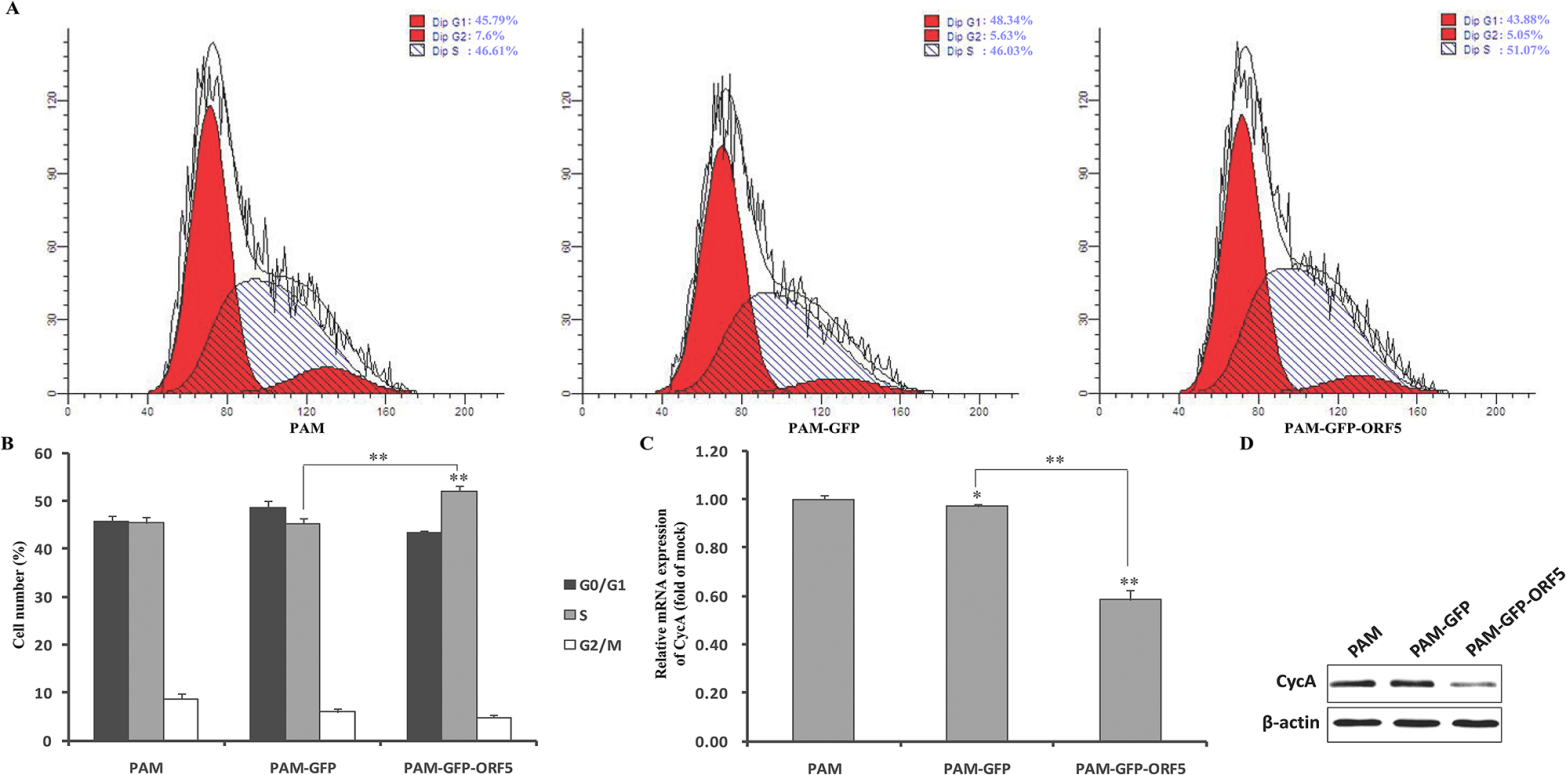
Analysis of the stages of cell division of expressing GFP-ORF5 protein by flow cytometry and effect of GFP-ORF5 expression on the porcine CycA protein expression. (A) Histograms from flow cytometry data for propidium iodide (PI) staining. (B) Quantitative analysis of the percentage of cells in each phase of the cell cycle from flow cytometry data. (C) Total RNA was extracted from cells expressing either GFP alone, GFP-ORF5 or untransfected cells. Real-time PCR analysis of CycA mRNA levels were normalized to the corresponding CT value for porcine β-actin mRNA. (D) The level of CycA expression was determined by western blot with the mouse monoclonal antibodies against CycA in all cell lines. β-actin was used as an internal loading control. The results are mean ± SD from three independent experiments. *p < 0.05 and **P<0.01 versus the control group.

To investigate the influence of the PCV2 ORF5 protein on cell growth, we confirmed PCV2 ORF5 protein expression in PAMs by fluorescence microscopy and western blot. As shown in Fig 5, PAMs transfected with pEGFP-ORF5 and pEGFP-C1 plasmids expressed proteins of approximately 33.5 kDa and 27 kDa, respectively, which was in good agreement with the predicted size of the ORF5 protein. As expected, the negative control PAM cells did not produce any immunostaining signal. Thereafter, the proliferation of GFP-ORF5-expressing cells and control cells was measured by CCK8 assay. As shown in Fig 6, the GFP-ORF5-expressing cells divided much more slowly compared to control cells, resulting in a significantly decreased cell number after a time course of 96 h. In a recent study, PCV2 replication has been found to be both S- and G2/M-phase dependent [38], indicating the possibility that the ORF5 protein might be involved in cell cycle progression. To validate this hypothesis, flow cytometric analysis was utilized to analyze the cell cycle via PI staining. As shown in Fig 7, the proportion of GFP-ORF5-expressing cells found to be in S-phase was 51.07%, whereas the proportions of the phase for the control cells were 46.03% and 46.61%, respectively. The results of further quantitative analysis of these data are displayed in Fig 7 and suggest that the presence of the GFP-tagged PCV2 ORF5 protein could trigger a higher proportion of cells to enter S-phase versus control cells. These findings suggest that the GFP-tagged PCV2 ORF5 protein prevents GFP-ORF5-expressing cells from entering the G2/M phase by prolonging the S-phase, resulting in cell growth inhibition.

To further elucidate the mechanism by which GFP-ORF5 induces prolongation of the S-phase cell cycle, qRT-PCR and western blot assays were employed to measure CycA mRNA and protein levels, as CycA is required for the entry into G2/M phase. As shown in Fig 7, both the transcription and translation of CycA were significantly down-regulated in pEGFP-ORF5-transfected cells relative to control cells. These results imply that both the inhibition of CycA transcription and the acceleration of CycA degradation may be responsible for GFP-ORF5-induced S-phase prolongation and further support a previous report that suggests that CycA is an important regulator of the PCV2 life cycle [38].

### The GFP-tagged PCV2 ORF5 protein has no effect on cell apoptosis

**Fig 8:**
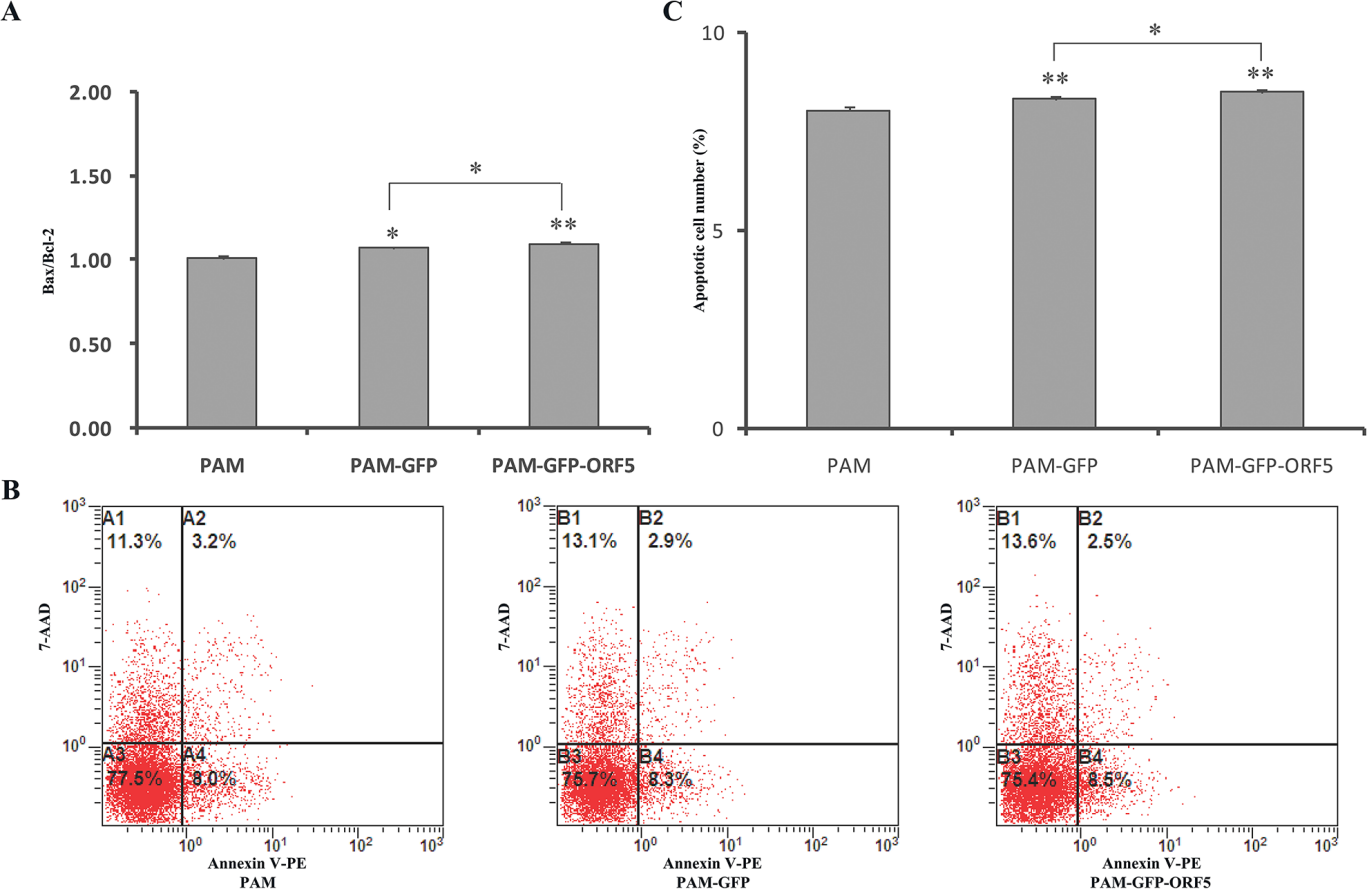
Effect of the GFP-tagged PCV2 ORF5 on the PAM apotosis. (A) Effect of PCV2 ORF5 on the mRNA expression of Bax and Bcl-2 genes. Real-time PCR analysis of Bax and Bcl-2 mRNA levels were normalized to the corresponding CT value for porcine β-actin mRNA, and the ratioes of Bax and Bcl-2 were used to generate histograms. (B) The cell apoptosis analysis in PAMs with flow cytometry. (C) Quantitative analysis of the percentage of apoptotic cells from flow cytometry data. The results are mean ± SD from three independent experiments. *p < 0.05 and **P<0.01 versus the control group.

As discussed above, the ability of the ORF5 protein to interact with either ORF3 and/or ORF4 is unclear. Considering the remarkable effects of ORF3 and ORF4 on PCV2-induced apoptosis, it is conceivable that the ORF5 protein also plays a role in apoptosis. To address this question, the effect of the ORF5 protein on apoptosis was assessed in PAMs via qRT-PCR and flow cytometric analysis. As shown in Fig 8, cells expressing the GFP-ORF5 protein did not produce any obvious changes in apoptotic activity compared to control cells after a period of 48 h, indicating that GFP-ORF5 expression does not affect cell death.

### The GFP-tagged PCV2 ORF5 protein localizes to the endoplasmic reticulum (ER) and induces ER stress

**Fig 9:**
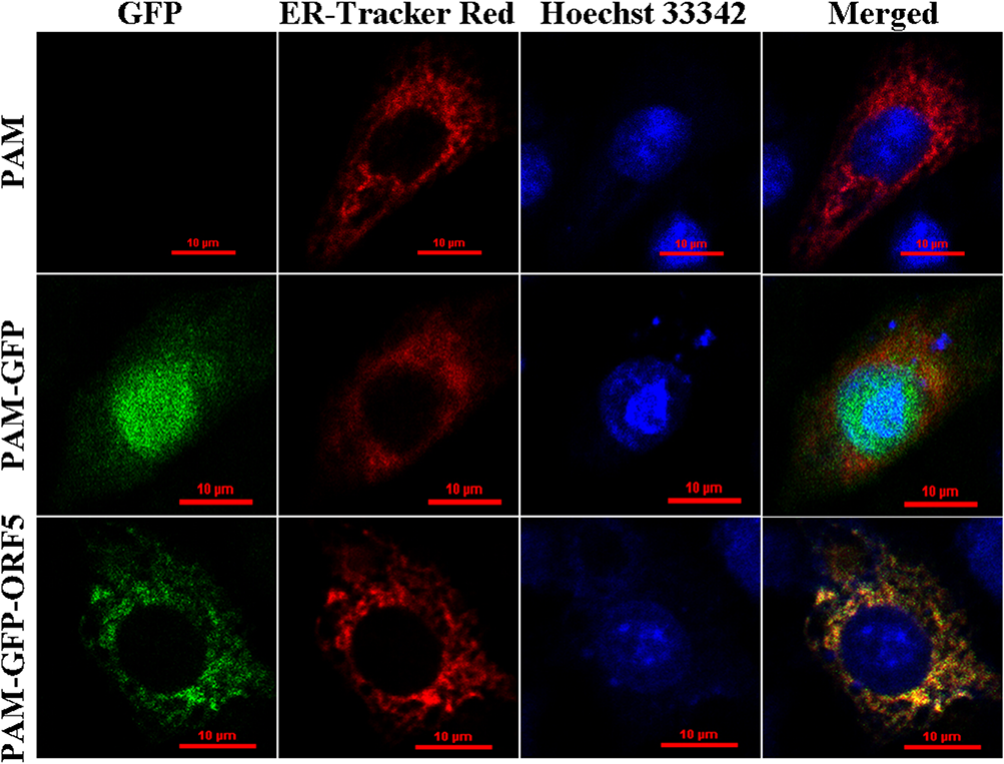
Detection of the subcellular localization of the GFP-ORF5 protein in PAMs by confocal microscopy. All the cells were stained by Hoechst33342 and ER-Tracker Red. Merged images showed co-localization of GFP-ORF5 protein in the ER. Bars, 10 μm.

**Fig 10:**
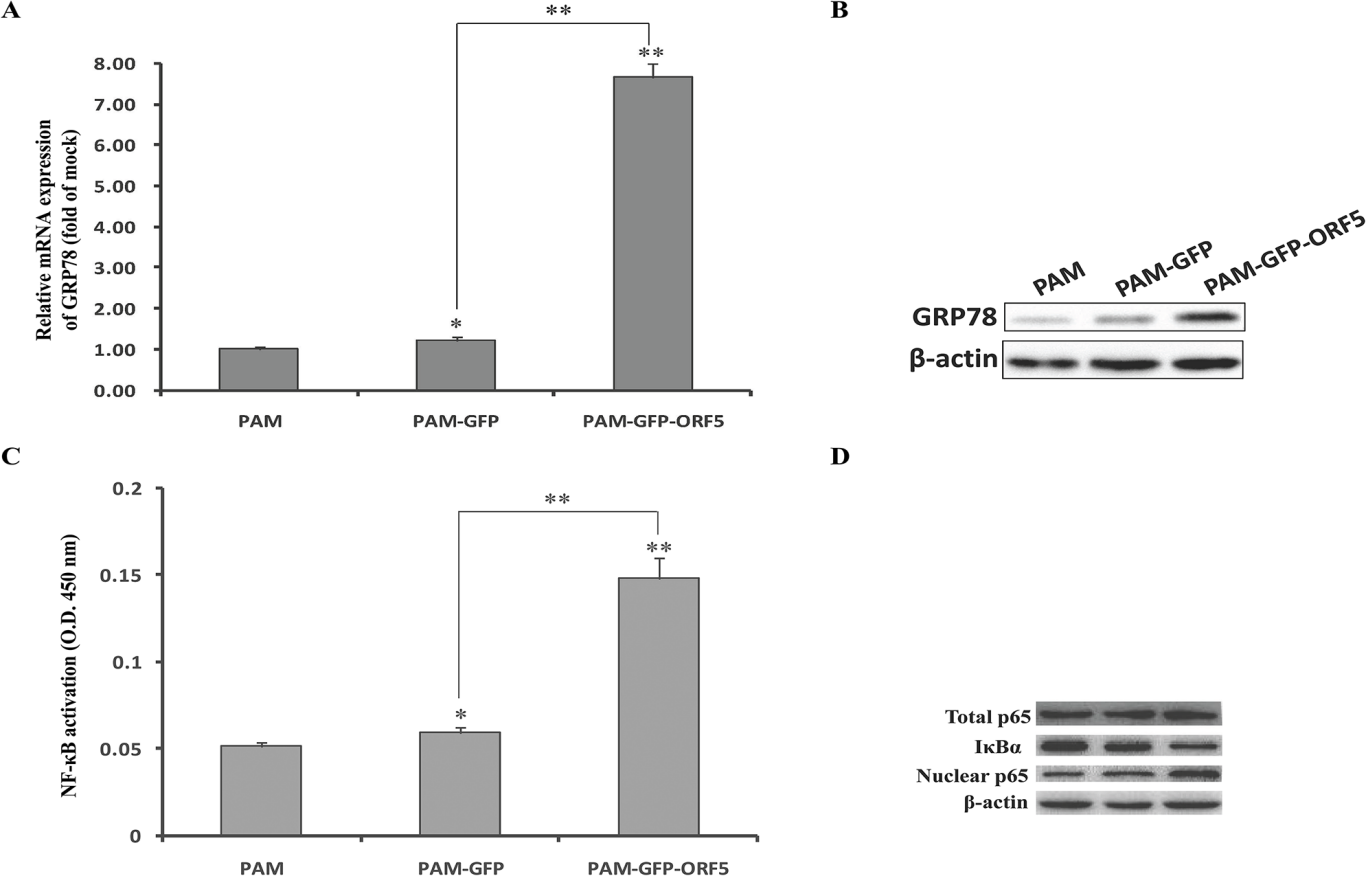
The GFP-tagged PCV2 ORF5 induces ER stress in PAMs. (A) Total RNA was extracted from cells expressing either GFP alone, GFP-ORF5 or untransfected cells. Real-time PCR analysis of GRP78 mRNA levels was normalized to the corresponding CT value for porcine β-actin mRNA. The results are mean ± SD and representative of three independent experiments. (B) The level of GRP78 expression was determined by western blot with the mouse monoclonal antibodies against GRP78. β-actin was used as an internal loading control. (C) NF-κB p65 activation was determined using the TransAM assay. The data represent the mean and standard deviation from three different experiments. (D) Cellular extracts were subjected to western blot analysis with antibodies specific for endogenous IκBα, NF-κB p65 and total p65, and anti-β-actin was used as a control for sample loading.

Confocal fluorescence microscopy was utilized to determine the subcellular localization of the ORF5 protein. As shown in Fig 9, the GFP-ORF5 protein localized to the endoplasmic reticulum (ER), whereas the GFP protein was predominantly located in the nucleus and to a lesser degree in the cytoplasm. Due to the ER localization of the GFP-ORF5 protein, the possibility that GFP-ORF5 induces ER stress must be assessed. To accomplish this, we first examined the expression level of glucose regulated protein 78 (GRP78), which is a well-characterized ER chaperone protein and a marker of ER stress [46]. As shown in Fig 10, GRP78 expression was significantly up-regulated at both the transcriptional and translational levels in GFP-ORF5-expressing cells compared with controls, suggesting that the GFP-ORF5 protein has a likely role in ER stress activation. To further support these findings, NF-κB activity was detected using ELISA. As shown in Fig 10, the activity of NF-κB in GFP-ORF5-expressing cells was significantly higher than in control cells, which confirmed the conclusion that the GFP-ORF5 protein could induce ER stress. To uncover the underlying mechanism by which GFP-ORF5 activates NF-κB, we surveyed the expression levels of IκBα, nuclear p65 and total p65 by western blot assay. As shown in Fig 10, the amount of the IκBα protein measured from PAMs transfected with pEGFP-ORF5 decreased obviously while the amount of the nuclear p65 protein increased considerably and the total p65 remained constant. These findings indicated that GFP-ORF5 induces NF-κB activation by promoting degradation of IκBα and nuclear translocation of p65.

### The GFP-tagged PCV2 ORF5 protein stimulates the upregulation of NF-κB-mediated downstream genes

**Fig 11:**
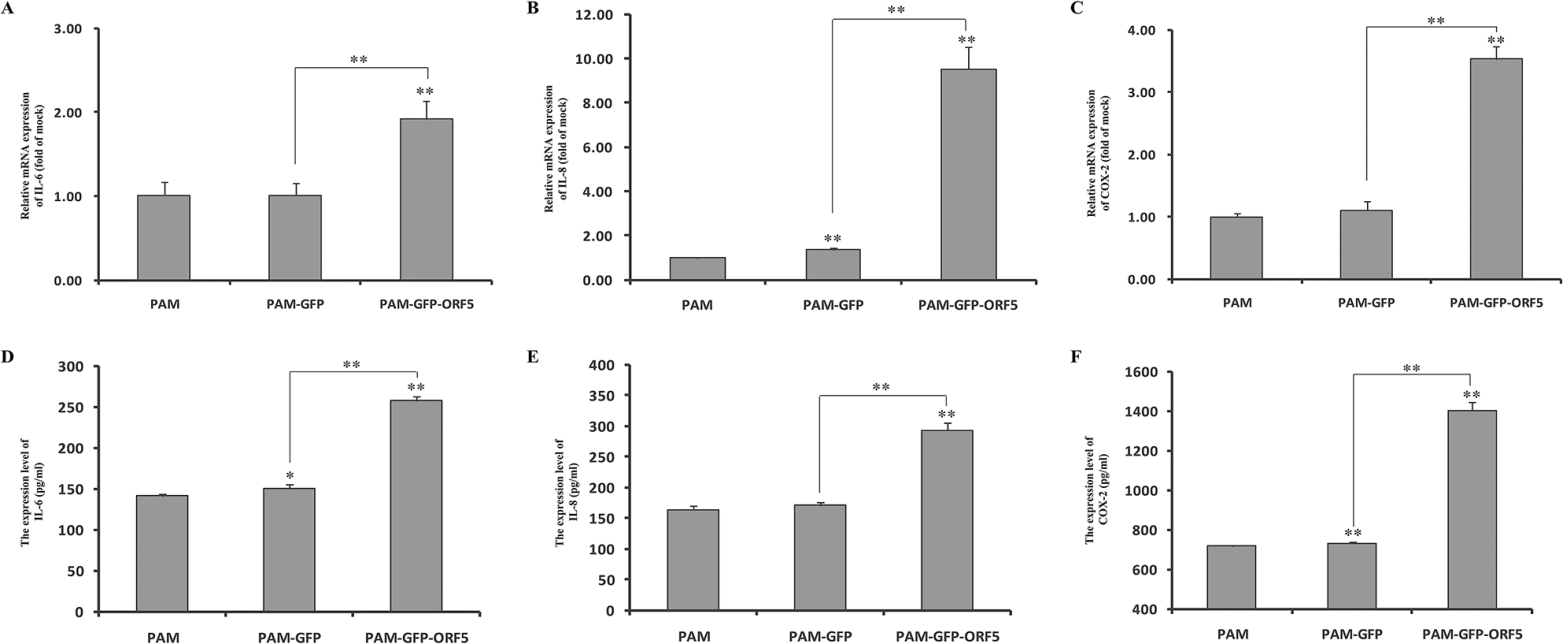
The GFP-tagged PCV2 ORF5 up-regulates IL-6, IL-8 and COX-2 expression in PAMs. PAMs were transfected with pEGFP-C1 or pEGFP-ORF5 plasmids and harvested at 48 h post-transfection. (A to C) The effect of PCV2 ORF5 expression on porcine IL-6, IL-8 and COX-2 transcription in cultured PAMs. Total RNA was extracted from cells expressing either GFP alone, GFP-ORF5 fusion, or untransfected cells. Realtime RT-PCR analysis of IL-6, IL-8 and COX-2 mRNA levels were normalized to the corresponding CT value for porcine β-actin mRNA. The results are mean ± SD and representative of three independent experiments. (D to F) The concentrations of IL-6, IL-8 and COX-2 in GFP-ORF5 expressing PAMs or control cells culture supernatants were measured by ELISA. Data are mean ± SD and representative of three independent experiments. *p < 0.05 and **P<0.01 versus the control group.

It is well known that the inflammatory factors IL-6, IL-8, and COX-2 are tightly regulated by activation of NF-κB. As the GFP-tagged PCV2 ORF5 protein could activate NF-κB, the expression of these factors might therefore be up-regulated. To clarify this matter, transcriptional levels of IL-6, IL-8, and COX-2 were measured using qRT-PCR. As shown in Fig 11, mRNA levels of IL-6, IL-8, and COX-2 in GFP-ORF5 expressing cells were significantly higher than in control cells. The secretion of the above listed factors into the culture media of transfected and untransfected cells was also examined by ELISA. As shown in Fig 11, the expression of GFP-ORF5 promoted a relative upregulation of the expression of IL-6, IL-8, and COX-2 compared to control cells. These results suggested that GFP-ORF5 up-regulates IL-6, IL-8, and COX-2 expression in PAMs. As it has been demonstrated that the overexpression of porcine CD74 can also promote the transcription of NF-κB-regulated genes [18], we therefore speculated that the ORF5 protein produces a synergistic effect with porcine CD74 on modulating the NF-κB signaling pathway during the process of PCV2 infection.

### Degradation of the GFP-tagged PCV2 ORF5 protein occurs via stimulation of proteasomal activity

**Fig 12:**
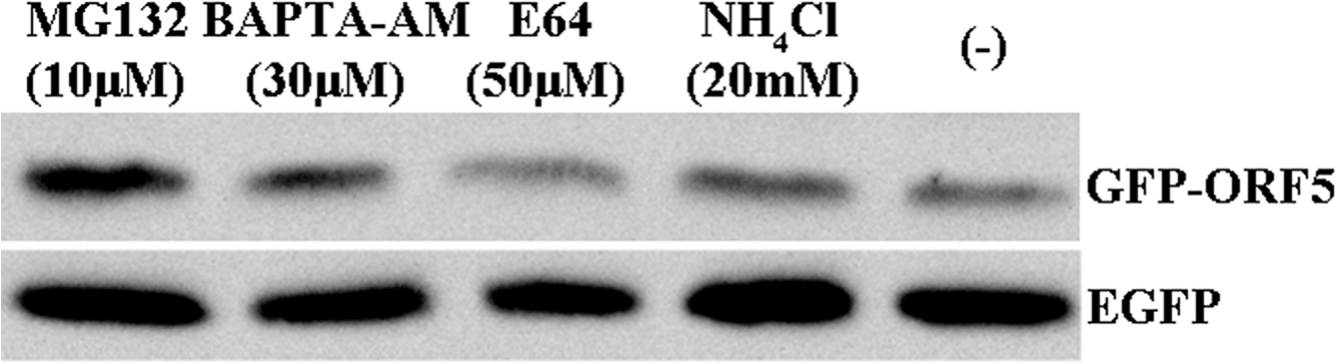
Proteasome inhibitors block the degradation of GFP-ORF5 protein. PAMs transfected with pEGFP-C1 or pEGFP-ORF5 were treated with 10 μM MG132, or with 30 μM BAPTA-AM, 50 μM E64, or 20 mM NH_4_Cl starting at 24 h after transfection. The cells were collected 12 h later and GFP-ORF5 proteins were detected by immunoblotting using anti-GFP antibody. EGFP was used as a control.

As the intensity of green fluorescence signal of GFP-ORF5 markedly decreased at 48 h post transfection, it is possible that the ORF5 protein undergoes degradation by the host cell during PCV2 infection. To investigate the degradation characteristics of the ORF5 protein, we first assessed the effect of proteasome inhibition on this process. Cells that had been transfected with either pEGFP-ORF5 or pEGFP-C1 were treated with the proteasome inhibitor MG132 and the expression levels of GFP-ORF5 and GFP proteins were analyzed by immunoblot analysis. As shown in Fig 12, GFP-ORF5 protein degradation was restrained effectively by the treatment, whereas no such change was observed for GFP protein. Furthermore, no protective effect was conferred by the lysosomal protease inhibitor E64, the lysosomal acidification inhibitor NH_4_Cl or the calcium chelator BAPTA-AM. Thus, these data indicated that the facilitated degradation of GFP-ORF5 protein is through a mechanism involving the proteasomal pathway.

### Identification of cellular proteins that interact with the PCV2 ORF5 protein

**Fig 13:**
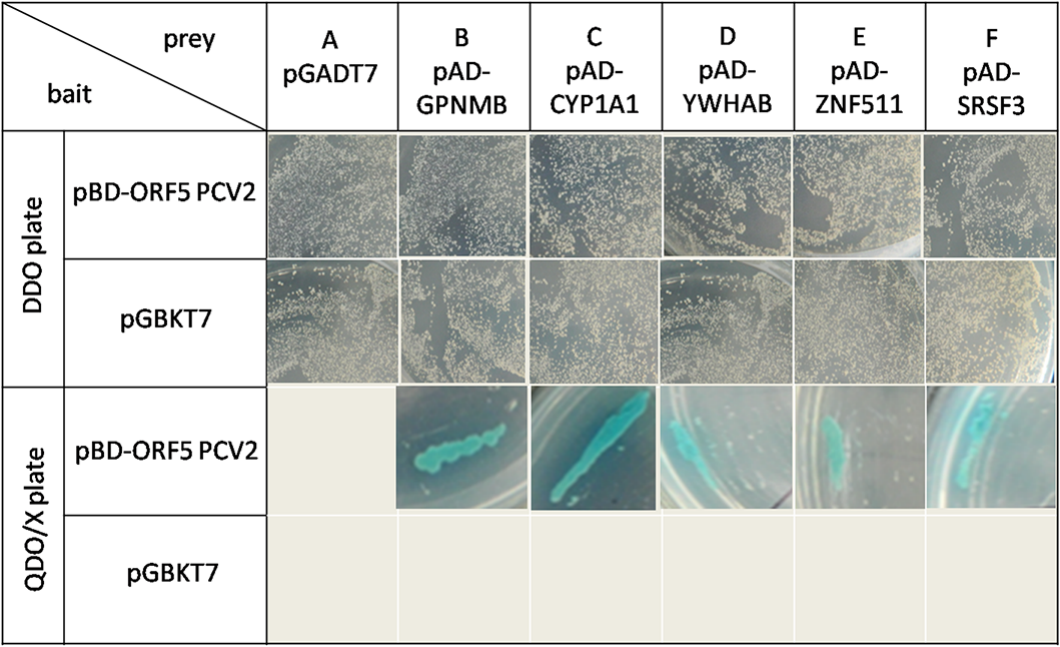
Yeast two-hybrid assay with the PCV2 ORF5 protein and host proteins. Yeast strain Y2H was co-transformed with pGBKT7-based bait and pGADT7-based prey plasmids and selected for the presence of both plasmids on DDO plates lacking tryptophane and leucine. Interaction of the ORF5 protein with the five cellular proteins GPNMB, CYP1A1, YWHAB, ZNF511 and SRSF3 was indicated by growth of blue colonies on QDO/X plates.

To identify cellular proteins that interact with the ORF5 protein of PCV2, ORF5 was used as bait in a yeast two-hybrid approach to screen a cDNA library derived from the porcine alveolar macrophages of a healthy pig. A total number of 16 positive clones were rescued, sequenced and analyzed by a BLAST search for homology to human and porcine proteins. To confirm the results, the bait constructs were co-transformed into the Y2H yeast strain, along with prey plasmids encoding putative interacting partners. After screening the co-transformation on double dropout (DDO) plates, quadruple dropout plus X-α-Gal (QDO/X) plates were used to confirm physical interactions between identified protein pairs. Table 2 lists the porcine proteins found to interact with the PCV2 ORF5 protein and indicates the frequency of the clones. As shown in Fig 13, the five cellular proteins found to interact with the ORF5 protein are the transmembrane glycoprotein NMB precursor (GPNMB), the cytochrome P450, family 1, subfamily A, polypeptide 1 (CYP1A1), the 14-3-3 protein beta/alpha (YWHAB), the zinc finger protein 511 isoform X2 (ZNF511), and the serine/arginine-rich splicing factor 3 (SRSF3).

**Table 2:** Identified cDNA clones coding for PCV2 ORF5 interacting proteins.

Number of cDNA clones found	Description of cDNA clones and best hits of homolog proteins identified in databases
5	Partial cDNA encoding aa 2–208 of porcine transmembrane glycoprotein NMB precursor (*Sus scrofa* NP_001092054.1, E value = 2e-69; *Homo sapiens* NP_001005340.1, E value = 1e-49)
4	Partial cDNA encoding aa 4–283 of porcine cytochrome P450 1A1 (*Sus scrofa* NP_999577.1, E value = 0; *Homo sapiens* NP_000490.1, E value = 7e-170)
1	Partial cDNA encoding aa 1–229 of porcine 14-3-3 protein beta/alpha (*Sus scrofa* XP_005673018.1, E value = 7e-166; *Homo sapiens* NP_003395.1, E value = 8e-166)
4	Partial cDNA encoding aa 7–38 of ZNF511 similar to porcine zinc finger protein 511 isoform X2 (*Sus scrofa* XP_005652477.1, E value = 0.002)
2	Partial cDNA encoding aa 1–101 of SRSF3 with similarity to serine/arginine-rich splicing factor 3 of human (*Homo sapiens* NP_003008.1, E value = 2e-67)

## Discussion

Many recent inquiries have focused on studying the gene sequence of PCV2. Despite this, both the total number of viral proteins encoded by PCV2 and the potential functions of these novel proteins have not yet been determined. In this study, a novel transcript ORF5 RNA could be easily detected by RT-PCR in PCV2-infected PAMs and further detected by northern blot assay (Fig 1). At 180 bp in length, this transcript was smaller than most confirmed viral RNAs but similar to the newly identified ORF4. Here, we demonstrated that ORF5 transcription was initiated during the process of PCV2 replication. Furthermore, to validate whether the ORF5 transcript can be translated into an actual protein by PCV2, we generated a specific mouse polyclonal antibody against the ORF5 protein. Immunofluorescence showed that the putative ORF5 protein of PCV2 was indeed expressed during PCV2 infection and was predominantly located in the cytoplasm of the infected cells (Fig 2). It is unfortunate, however, that the ORF5 protein signal could not be detected via western blots in protein samples that were treated with loading buffer and boiled. Previous studies have reported that some viral proteins are only short-lived and sharply decay in cells, such as CSFV NS2 protein and HCV p7 protein [47–48]. Thus, we infer that the polypeptide encoded by *ORF5-RNA* may be either expressed at low levels and transiently during PCV2 infection or that its antigenic epitopes were seriously destroyed during post-harvest processing. However, if possible, the natural existence of ORF5 protein during PCV2 infection needs to be further confirmed by independent experiments.

Further investigation indicated that ORF5-deficient PCV2 replicates slowly in PAMs, whereas the maximal DNA copies and viral titers are comparable to wPCV2, a phenomenon similar to that of ORF3-deficient PCV2 [21]. Hence, certain features or potencies of the ORF3 protein may also be suitable for ORF5. Because it has been suggested that ORF3 could enhance the spread of PCV2, and that abrogation of ORF3 will attenuate its virulence [28], it will also be meaningful to analyze the growth characteristic of PCV2Δ in its natural host. Such studies will be beneficial toward deepening our understanding of the role played by this protein and its potential role in creating a vaccine strain that could elicit an appropriate immune response against wild-type PCV2. Careful analysis of the transcriptional curves of each gene suggests that the lack of ORF5 protein expression blocks the transcription of ORF1 and ORF2, whereas ORF5 does not have any impact on the transcription of ORF3 and ORF4 (Fig 4). Previous studies have demonstrated that ORF3 promotes apoptosis, whereas ORF4 interferes with the process. These data would imply that the ORF5 protein might not be implicated in PCV2-induced apoptosis. Additional investigations into the effect of the ORF5 protein on apoptosis via qRT-PCR and flow cytometric analysis also confirmed this claim (Fig 8).

The S-phase plays a critical role in viral replication during cell cycle progression. Many viruses manipulate the S-phase of the cell cycle to create a cellular environment that is beneficial for their own replication, including pestiviruses (CSFV NS2 protein and HCV NS2 protein) and coronaviruses (PEDV N protein and SARS-CoV N protein) [44, 49–51]. However, whether PCV2, one type of circoviruses, can induce cell cycle arrest at a particular phase is not clear. Similarly, our findings in this study showed that the GFP-tagged PCV2 ORF5 protein is also able to inhibit cell proliferation (Fig 6) and prolong the S-phase of the cell cycle via the transcriptional inhibition and accelerated degradation of CycA (Fig 7), indicating that the PCV2 ORF5 protein may regulate CycA expression. Interestingly, a recent study demonstrated that PCV2 replication is both S- and G2/M-phase dependent and that CycA expression levels play an important role during the PCV2 life cycle [38]. Therefore, it is assumed that the PCV2 ORF5 protein may contribute to PCV2 replication by regulating CycA expression.

As the subcellular localization of a protein will usually reflect its function [45], we examined the localization of ORF5 using confocal fluorescence microscopy. We demonstrated that the GFP-tagged PCV2 ORF5 protein localizes to the ER (Fig 9) and is able to induce ER stress via the upregulation of both the molecule chaperon GRP78 and nuclear factor kappa B (NF-κB) (Fig 10). NF-κB is considered to be an important regulator of the expression of various genes during viral infection, particularly with regard to the secretion of the proinflammatory factors IL-6, IL-8, and COX-2 [18]. Thus, the activation of NF-κB is considered to be a protective response [52]. Our findings show that overexpression of the GFP-tagged PCV2 ORF5 protein elevates NF-κB activity by promoting IκBα degradation and p65 nuclear translocation and is consistent with previous results demonstrating that PCV2 infection activates the NF-κB signalling pathway in PK-15 cells [53]. Collectively, these results imply that PCV2 ORF5 may enhance viral replication, survival, and even immune evasion. Furthermore, the upregulation of IL-6, IL-8 and COX-2 (Fig 11) following overexpression of GFP-ORF5 further confirmed this hypothesis. Thus, these results suggest that PCV2 ORF5 probably palys a critical role in assisting PCV2 infection by regulating the NF-κB signaling pathway, leading to an enhanced inflammatory response.

As PCV2 is the smallest DNA virus known to infect mammals, its genome is very compact and has a highly limited coding capacity. Thus, PCV2 replication depends extensively on host cellular factors. Therefore, it is generally acknowledged that PCV2 must not only interact with the host cell to subvert cellular factors or processes to complete viral replication but also to modulate host immunity to cause cytokine imbalance, immunosuppression, and disease, utilizing its DNA sequences or encoded proteins. Therefore, the identification of cellular proteins that interact with PCV2 viral factors and the detailed description of such interactions are of paramount importance. In this study, five cellular proteins including type I transmembrane glycoprotein (GPNMB), cytochrome P450 enzyme (CYP1A1), adapter protein (YWHAB), transcriptional regulator (ZNF511) and RNA splicing factor (SRSF3), were identified to interact with the PCV2 ORF5 protein (summarized in Table 2), although the domains responsible for these interactions and their biological significance remain largely unknown. Undoubtedly, further studies will be necessary to elucidate the exact molecular mechanisms governing virus-host interplay and will be very useful toward gaining further understanding of the dependency of viral growth on host cell factors, as well as the pathogenic mechanism of PCV2 infection.

Curiously, the previously detected NS0 RNAs, whose coding sequences (23 aa) encompass nucleotides 661–732 [26], are fully included within the ORF5 gene. Moreover, the relative expression level of ORF5 was more abundant than ORF1 (Fig 4), which is consistent with previous northern blot analysis that demonstrates that PCV2 NS0 is more abundant than PCV2 Rep [54]. To date, it is not clear whether the minor NS0 RNA encodes a protein or what function such a protein may possess during the life cycle of PCV2. Previous work has suggested that PCV2 NS0 is more abundant than PCV1 NS0 and that the differential splicing and relative expression levels of NS0 RNAs may contribute to the pathogenesis of PCV2 [54]. However, contradictory evidence has also shown that introducing a stop codon into NS0 RNA did not have any effect on viral protein synthesis or DNA replication [22]. In consideration of all of the above, we conclude that the alleged NS0 may be merely a fraction of ORF5 and is therefore incapable of playing as extensive of a role as the intact ORF5.

## Conclusion

In conclusion, ORF5 is a novel PCV2 protein that is not essential for viral replication in cultured PAMs and likely plays an important role in persistent PCV2 infection by regulating the NF-κB signaling pathway. Nevertheless, due to the inability to detect the putative PCV2 ORF5 protein by western blot and the possibility that functions of GFP-ORF5 perhaps not completely represent those of ORF5 alone, additional studies are required to validate the expression and function of the ORF5 protein during PCV2 infection in vitro.

## Supporting Information

S1 Fig Nucleotide sequence of PCV2 Yangling strain (wPCV2). Virally encoded proteins that have been determined are annotated in this sequence. RNAs transcribed to right (Rep, Rep' and ORF5) are annotated on top of the sequence, while the RNAs (Cap, ORF3 and ORF4) transcribed in the leftward orientation are annotated below the sequence. Initiation codons are in oval circles and termination codons are in boxes. The EcoRI restriction enzyme site is also indicated with an underscore.
(TIF)

## References

[1] Allan GM, Kennedy S, McNeilly F, Foster JC, Ellis JA, Krakowka SJ, et al Experimental reproduction of severe wasting disease by co-infection of pigs with porcine circovirus and porcine parvovirus. J Comp Pathol. 1999; 121:1–11. 10373289 10.1053/jcpa.1998.0295

[2] Kennedy S, Moffett D, McNeilly F, Meehan B, Ellis J, Krakowka S, et al Reproduction of lesions of postweaning multisystemic wasting syndrome by infection of conventional pigs with porcine circovirus type 2 alone or in combination with porcine parvovirus. J Comp Pathol. 2000; 122:9–24. 10627387 10.1053/jcpa.1999.0337

[3] Bolin SR, Stoffregen WC, Nayar GP, Hamel AL. Postweaning multisystemic wasting syndrome induced after experimental inoculation of cesarean-derived, colostrum-deprived piglets with type 2 porcine circovirus. J Vet Diagn Investig. 2001; 13:185–194. 11482594 10.1177/104063870101300301

[4] Allan GM, McNeilly F, Ellis J, Krakowka S, Botner A, McCullough K, et al PMWS: experimental model and co-infections. Vet Microbiol. 2004; 98:165–168. 14741129 10.1016/j.vetmic.2003.10.009

[5] Segalés J, Sitjar M, Domingo M, Dee S, Del Pozo M, Noval R, et al First report of postweaning multisystemic wasting syndrome in pigs in Spain. Vet Rec. 1997; 141:600–601. 9429277

[6] Chae C. Postweaning multisystemic wasting syndrome: a review of aetiology, diagnosis and pathology. Vet J. 2004; 168:41–49. 15158207 10.1016/j.tvjl.2003.09.018

[7] Darwich L, Segales J, Mateu E. Pathogenesis of postweaning multisystemic wasting syndrome caused by Porcine circovirus 2: An immune riddle. Arch Virol. 2004; 149:857–874. 15098103 10.1007/s00705-003-0280-9

[8] Segales J, Domingo M. Postweaning multisystemic wasting syndrome (PMWS) in pigs: a review. Vet Q. 2002; 24:109–124. 12400999 10.1080/01652176.2002.9695132

[9] Allan GM, Ellis JA. Porcine circoviruses: a review. J Vet Diagn Invest. 2000; 12:3–14. 10690769 10.1177/104063870001200102

[10] Segales J, Rosell C, Domingo M. Pathological findings associated with naturally acquired porcine circovirus type 2 associated diseases. Vet Microbiol. 2004; 98:137–149. 14741126 10.1016/j.vetmic.2003.10.006

[11] Chae C. A review of porcine circovirus 2 associated syndromes and diseases. Vet J. 2005; 169:326–336. 15848776 10.1016/j.tvjl.2004.01.012

[12] Opriessnig T, Meng XJ, Halbur PG. Porcine circovirus type 2 associated diseases: update on current terminology, clinical manifestations, pathogenesis, diagnosis, and intervention strategies. J Vet Diagn Invest. 2007; 19 (6): 591–615. 17998548 10.1177/104063870701900601

[13] Ramamoorthy S, Meng XJ. Porcine circoviruses: a minuscule yet mammoth paradox. Anim Health Res Rev. 2009; 10 (1):1–20. 10.1017/S1466252308001461 18761774

[14] Gillespie J, Opriessnig T, Meng XJ, Pelzer K, Buechner-Maxwell V. Porcine circovirus type 2 and porcine circovirus associated diseases. J Vet Intern Med. 2009; 23(6):1151–1163. 10.1111/j.1939-1676.2009.0389.x 19780932 PMC7166794

[15] Segalés J. Porcine circovirus type 2 (PCV2) infections: clinical signs, pathology and laboratory diagnosis. Virus Res. 2012; 164:10–19. 10.1016/j.virusres.2011.10.007 22056845

[16] Chae C. Porcine circovirus type 2 and its associated diseases in Korea. Virus Res. 2012; 164:107–113. 10.1016/j.virusres.2011.10.013 22027190

[17] Meng XJ. Porcine circovirus type 2 (PCV2): Pathogenesis and Interaction with the Immune System. Annu Rev Anim Biosci. 2013; 1:43–64. 10.1146/annurev-animal-031412-103720 25387012

[18] Zhang HL, Liu C, Cheng S, Wang XF, Li WT, Charreyre C, et al Porcine CD74 is involved in the inflammatory response activated by nuclear factor kappa B during porcine circovirus type 2 (PCV-2) infection. Arch Virol. 2013; 158(11):2285–2295. 10.1007/s00705-013-1750-3 23736979

[19] Hamel AL, Lin LL, Nayar GPS. Nucleotide sequence of porcine circovirus associated with postweaning multisystemic wasting syndrome in pigs. J Virol. 1998; 72:5262–5267. 9573301 10.1128/jvi.72.6.5262-5267.1998 PMC110114

[20] Guo L, Lu Y, Wei Y, Huang L, Liu C. Porcine circovirus type 2 (PCV2): genetic variation and newly emerging genotypes in China. Virol J. 2010; 7:273 10.1186/1743-422X-7-273 20955622 PMC2967541

[21] Liu J, Chen I, Kwang J. Characterization of a previously unidentified viral protein in porcine circovirus type 2-infected cells and its role in virus-induced apoptosis. J Virol. 2005; 79:8262–8274. 15956572 10.1128/JVI.79.13.8262-8274.2005 PMC1143760

[22] Cheung AK. The essential and nonessential transcription units for viral protein synthesis and DNA replication of porcine circovirus type 2. Virology 2003; 313:452–459. 12954212 10.1016/s0042-6822(03)00373-8

[23] Nawagitgul P, Morozov I, Bolin SR, Harms PA, Sorden SD. Open reading frame 2 of porcine circovirus type 2 encodes a major capsid protein. J Gen Virol. 2000; 81:2281–2287. 10950986 10.1099/0022-1317-81-9-2281

[24] He JL, Cao JJ, Zhou N, Jin YL, Wu JS, Zhou JY. Identification and functional analysis of the novel ORF4 protein encoded by porcine circovirus type 2. J Virol. 2013; 87(3):1420–1429. 10.1128/JVI.01443-12 23152517 PMC3554175

[25] Gao ZZ, Dong QF, Jiang YH, Opriessnig T, Wang JX, Quan YP, et al ORF4-protein deficient PCV2 mutants enhance virus-induced apoptosis and show differential expression of mRNAs in vitro. Virus Res. 2014; 183:56–62. 10.1016/j.virusres.2014.01.024 24503223

[26] Cheung AK. Transcriptional analysis of porcine circovirus type 2. Virology 2003; 305:168–180. 12504550 10.1006/viro.2002.1733

[27] Mankertz A, Mankertz J, Wolf K, Buhk HJ. Identification of a protein essential for replication of porcine circoirus. J Gen Virol. 1998; 79:381–383. 9472624 10.1099/0022-1317-79-2-381

[28] Karuppannan AK, Jong MH, Lee SH, Zhu Y, Selvaraj M, Lau J, et al Attenuation of porcine circovirus 2 in SPF piglets by abrogation of ORF3 function. Virology 2009; 383:338–347. 10.1016/j.virol.2008.10.024 19012942

[29] Meehan BM, Creelan JL, McNulty MS, Todd D. Sequence of porcine circovirus DNA: affinities with plant circoviruses. J Gen Virol. 1997; 78:221–227. 9010307 10.1099/0022-1317-78-1-221

[30] Zhou JY, Chen QX, Ye JX, Shen HG, Chen TF, Shang SB. Serological investigation and genomic characterization of PCV2 isolates from different geographic regions of Zhejiang province in China. Vet Res Commun. 2006; 30:205–220. 16400605 10.1007/s11259-006-3203-x

[31] Allan GM, McNeilly F, Cassidy JP, Reilly GA, Adair B, Ellis WA, et al Pathogenesis of porcine circovirus: experimental infections of colostrums deprived piglets and examination of pig foetal material. Vet Microbiol. 1995; 44:49–64. 7667906 10.1016/0378-1135(94)00136-k

[32] Tischer I, Mields W, Wolff D, Vagt M, Griem W. Studies on epidemiology and pathogenicity of porcine circovirus. Arch Virol. 1986; 91:271–276. 3778212 10.1007/BF01314286

[33] Gilpin DF, McCullough K, Meehan BM, McNeilly F, McNair I, Stevenson LS, et al In vitro studies on the infection and replication of porcine circovirus type 2 in cells of the porcine immune system. Vet Immunol Immunop. 2003; 94(3–4):149–161. 10.1016/s0165-2427(03)00087-4 12909411

[34] Chang HW, Pang VF, Chen LJ, Chia MY, Tsai YC. Bacterial lipopolysaccharide induces porcine circovirus type 2 replication in swine alveolar macrophages. Vet Microbiol. 2006; 115(4):311–319. 16626898 10.1016/j.vetmic.2006.03.010

[35] Choi C, Chae C. Distribution of porcine parvovirus in porcine circovirus 2-infected pigs with postweaning multisystemic wasting syndrome as shown by in-situ hybridization. J Comp Pathol. 2000; 123(4):302–305. 11042001 10.1053/jcpa.2000.0421

[36] McGuire K, Glass EJ. The expanding role of microarrays in the investigation of macrophage responses to pathogens. Vet Immunol Immunop. 2005; 105(3):259–275. 10.1016/j.vetimm.2005.02.001 15808305

[37] Tang ZH, Guo KK, Zhang YM, Wang JJ, Li YL. Isolation and identification of porcine circovirus type 2 Yangling isolate and sequence analysis of the whole genomes. J Northwest A&F Univ (Nat. Sci. Ed.). 2011; 39(4):7–13.

[38] Tang QH, Li SB, Zhang H, Wei YW, Wu HL, Liu JB, et al Correlation of the cyclin A expression level with porcine circovirus type 2 propagation efficiency. Arch Virol. 2013; 158:2553–2560. 10.1007/s00705-013-1785-5 23836398

[39] Weingartl HM, Sabara M, Pasick J, van Moorlehem E, Babiuk L. Continuous porcine cell lines developed from alveolar macrophages: partial characterization and virus susceptibility. J Virol Methods. 2002; 104:203–216. 12088830 10.1016/S0166-0934(02)00085-X PMC7119708

[40] Livak KJ, Schmittgen TD. Analysis of relative gene expression data using real-time quantitative PCR and the 2-[Delta][Delta] CT Method. Methods 2001; 25(4):402–408. 11846609 10.1006/meth.2001.1262

[41] Mankertz A, Mueller B, Steinfeldt T, Schmitt C, Finsterbusch T. New Reporter Gene-Based Replication Assay Reveals Exchangeability of Replication Factors of Porcine Circovirus Types 1 and 2. J Virol. 2003; 77(18):9885–9893. 12941898 10.1128/JVI.77.18.9885-9893.2003 PMC224580

[42] Tischer I, Peters D, Rasch R, Pociuli S. Replication of porcine circovirus: induction by glucosamine and cell cycle dependence. Arch Virol. 1987; 96:39–57. 3619654 10.1007/BF01310989

[43] Reed LJEA. A simple method of estimating fifty percent endpoints. Am J Hyg. 1938; 27:493–497.

[44] Tang QH, Zhang YM, Fan L, Tong G, He L, Dai C. Classic swine fever virus NS2 protein leads to the induction of cell cycle arrest at S-phase and endoplasmic reticulum stress. Virol J. 2010; 7(1):4. 20064240 10.1186/1743-422X-7-4 PMC2819037

[45] Finsterbusch T, Steinfeldt T, Doberstein K, Rödner C, Mankertz A. Interaction of the replication proteins and the capsid protein of porcine circovirus type 1 and 2 with host proteins. Virology 2009; 386:122–131. 10.1016/j.virol.2008.12.039 19178923

[46] Lee AS. The ER chaperone and signaling regulator GRP78/BiP as a monitor of endoplasmic reticulum stress. Methods 2005; 35(4):373–381. 15804610 10.1016/j.ymeth.2004.10.010

[47] Guo KK, Zhang YM, Tang QH, Ning PB, Li HL, Lv QZ, et al Pilot study on degradation of classical swine fever virus nonstructural 2 protein in cells. J Anim Vet Adv. 2013; 12(2): 234–241.

[48] Haqshenas G. The p7 protein of hepatitis C virus is degraded via the proteasome-dependent pathway. Virus Res. 2013; 176:211–215. 10.1016/j.virusres.2013.06.009 23830937

[49] Xu XG, Zhang HL, Zhang Q, Huang Y, Dong J, Liang YB, et al Porcine epidemic diarrhea virus N protein prolongs S-phase cell cycle, induces endoplasmic reticulum stress, and up-regulates interleukin-8 expression. Vet Microbiol. 2013; 164:212–221. 10.1016/j.vetmic.2013.01.034 23562137 PMC7117426

[50] Surjit M. The nucleocapsid protein of severe acute respiratory syndrome-coronavirus inhibits the activity of cyclin-cyclin-dependent kinase complex and blocks S phase progression in mammalian cells. J Biol Chem. 2006; 281(16):10669–10681. 16431923 10.1074/jbc.M509233200 PMC7995956

[51] Yang XJ, Liu J, Ye L, Liao QJ, Wu JG. HCV NS2 protein inhibits cell proliferation and induces cell cycle arrest in the S-phase in mammalian cells through down-regulation of cyclin A expression.Virus Res. 2006; 121:134–143. 16797769 10.1016/j.virusres.2006.02.004

[52] Tato CM, Hunter CA. Host-pathogen interactions: subversion and utilization of the NF-kappa B pathway during infection. Infect Immun. 2002; 70:3311–3317. 12065467 10.1128/IAI.70.7.3311-3317.2002 PMC128040

[53] Wei L, Kwang J, Wang J, Shi L, Yang B, Li Y, et al Porcine circovirus type 2 induces the activation of nuclear factor kappa B by IkappaBalpha degradation. Virology 2008; 378:177–184. 10.1016/j.virol.2008.05.013 18561971

[54] Cheung AK. Comparative analysis of the transcriptional patterns of pathogenic and nonpathogenic porcine circoviruses. Virology 2003; 310:41–49. 12788629 10.1016/s0042-6822(03)00096-5

