# Correction: Identification of Putative ORF5 Protein of Porcine Circovirus Type 2 and Functional Analysis of GFP-Fused ORF5 Protein

**DOI:** 10.1371/journal.pone.0134203

**Published:** 2015-07-28

**Authors:** Qizhuang Lv, Kangkang Guo, Han Xu, Tao Wang, Yanming Zhang

There is an error in the legend for Fig 1, "Genomic location of ORF5 gene and Detection of ORF5 transcripts by RT-PCR and Northern blot from PCV2-infected cells," panel D. Please see the complete, correct Fig 1 legend here.

**Fig 1:**
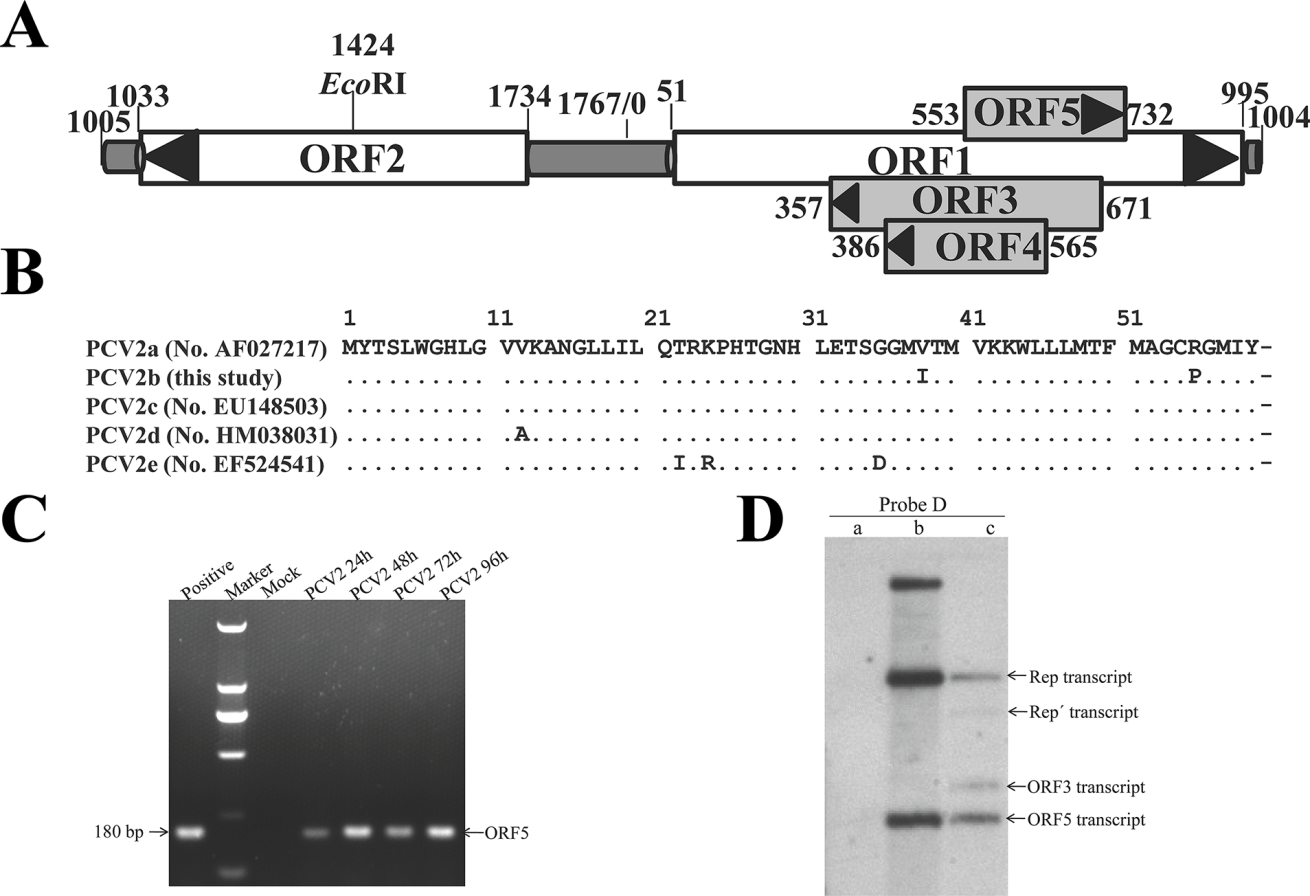
Genomic location of ORF5 gene and Detection of ORF5 transcripts by RT-PCR and Northern blot from PCV2-infected cells. A) Genomic schematic of PCV2 Yangling strain (wPCV2). Coding sequences of the five ORFs are annotated with nucleotide coordinates that indicate the nucleotide site of each gene. The ORF2, ORF3 and ORF4 genes are transcribed leftward, while the ORF1 and ORF5 genes are transcribed rightward. The EcoRI restriction enzyme site is also indicated. (B) Nucleotide and amino acid alignments of the putative ORF5 reported by Hamel et al in 1998 (pmws, GenBank accession no. AF027217 [19]) and our team (wPCV2, this study) using the ClustalV Method. Homologous nucleotides and amino acids are indicated by asterisks. (C) Analysis of the ORF5 gene in PCV2-infected cells by RT-PCR. RNA was isolated from PCV2- or mock-infected cells and copied into cDNA. The cDNA was amplified with a pair of ORF5 primers. Positive fragment was amplified from the PCV2 genome using PCR. (D) Northern blot identification of the ORF5 transcript in PAMs infected with wPCV2. Total RNA samples were isolated from mock- and wPCV2-infected PAMs. Lane a, no RNA signal was detected in mock-infected PAMs after hybridization with a DIG-labeled ORF5 DNA probe (probe D). Lane b, mixtures of pMD-19T-ORF1 (3625 bp), PCR products of ORF1 (945 bp) and ORF5 (180 bp) were hybridized with probe D. Lane c, Rep, Repˊ, ORF3 and ORF5 transcripts were hybridized with probe D in wPCV2-infected PAMs.
